# Hybrid Process of Oxidation and Separation of Organic Contaminants Using Copper(II) Complex-Decorated Nylon Membranes under Mild Conditions

**DOI:** 10.1021/acsomega.6c03603

**Published:** 2026-06-25

**Authors:** Felipe P. da Silva, Aline C. F. Pereira, Juliana C. Pinheiro, Annelise Casellato, Cristiano P. Borges, Fabiana V. da Fonseca

**Affiliations:** † Escola de Química, 28125 Universidade Federal do Rio de Janeiro, Av. Athos da Silveira Ramos 149, Bl. E, Cidade Universitária, Rio de Janeiro 21941-909, RJ, Brazil; ‡ Faculdade de Engenharia, Universidade do Estado do Rio de Janeiro, Rua São Francisco Xavier 524, Rio de Janeiro 20550-900, RJ, Brazil; § Instituto de Química, Universidade Federal do Rio de Janeiro, Av. Athos da Silveira Ramos 149, Bl. A, Cidade Universitária, Rio de Janeiro 21941-909, RJ, Brazil; ∥ Instituto Alberto Luiz Coimbra de Pós-Graduação e Pesquisa de Engenharia (COPPE), Universidade Federal do Rio de Janeiro, Av. Horácio Macedo 2030, Cidade Universitária, Rio de Janeiro 21941-972, RJ, Brazil

## Abstract

Industrial wastewater often contains recalcitrant organic contaminants that are difficult to remove, necessitating treatment strategies that integrate different mechanisms under relevant environmental conditions. In this study, a hybrid oxidation and separation process based on nylon membranes decorated with a copper­(II) (CuL) complex was developed at near-neutral pH and in the absence of light irradiation. The performance was evaluated in batch tests for the removal of the dye Drimaren Red X-6BN (DRX-6BN) and in filtration systems applied to the treatment of oily wastewater in the absence and presence of hydrogen peroxide (H_2_O_2_). The decorated membranes showed DRX-6BN removals of approximately 50% by adsorption in 300 min, while the addition of H_2_O_2_ increased the efficiency to values greater than 80% in 180 min. In filtration systems, the integration of catalytic oxidation and membrane separation resulted in the removal of up to ∼100% of the chemical oxygen demand of oily wastewater, even when using a membrane with the lowest CuL load. The activity of the material was maintained after storage for at least three months. These results provide initial evidence supporting the feasibility of CuL-decorated nylon membranes for advanced wastewater treatment applications.

## Graphical abstract

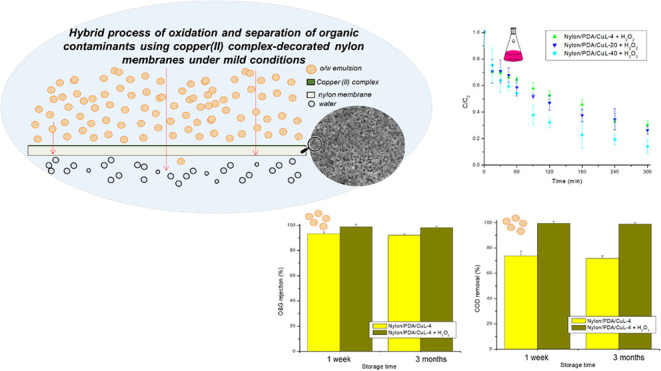

## 1 Introduction

Industrial activities generate aqueous wastewater containing mixtures of persistent organic pollutants, such as synthetic dyes and oils, which pose significant environmental risks owing to their toxicity, chemical stability, and resistance to biological degradation.^1–4^ Several approaches can be used for wastewater treatment, including coagulation/flocculation, chemical precipitation, biological and enzymatic treatments, adsorption, ion exchange, electrochemical techniques, advanced oxidation processes (AOP), and membrane separation processes (MSP).^5–12^ However, efficient wastewater treatment using approaches that combine multiple mechanisms, including adsorption, catalytic degradation, and separation, favors sustainable and practical remediation.^13^

Polymeric membranes have been extensively investigated for wastewater treatment because of their mechanical robustness, chemical stability, and ease of processing.^14–16^ However, conventional membranes operate predominantly through separation mechanisms, such as size exclusion, solubilization, and selective diffusion, in addition to electrostatic interactions,^17^ which limits their effectiveness against dissolved and recalcitrant organic pollutants because they do not promote the chemical transformation of the contaminants. The functionalization of membranes with catalytically active materials has emerged as a promising strategy to overcome these limitations by allowing the integration of separation and chemical transformation of contaminants in a single step. Surface modification with polydopamine (PDA) stands out in this scenario for providing a versatile platform, rich in functional groups, suitable for the stable immobilization of metal species, in addition to contributing to increased adsorptive affinity of the membrane.^18,19^

Despite the potential of membrane functionalization, the stable and homogeneous immobilization of catalytic species remains a significant challenge.^30^ Methods such as sol–gel, in situ growth, chemical grafting, and direct incorporation involve complex procedures, high solvent and energy consumption, limitations in catalyst loading, nonuniform distribution of active species, and risk of leaching during operation.^26,30^ These aspects reinforce the need for simple and versatile surface modification strategies capable of promoting efficient catalyst anchoring without compromising the membrane structure and performance.^18,19^

Among the catalysts used in AOP, copper­(II) complexes have relevant advantages, such as the ability to promote Fenton-like reactions under milder conditions, including near-neutral pH, in contrast to iron-based systems.^20–22^ In a previous report, the copper­(II) bis-*N*-(2-hydroxyethyl)­salicylaldiminate complex (CuL), the same one used in this study, showed 90.17 ± 0.52% efficiency in the degradation of the Drimaren Red X-6BN (DRX-6BN), at pH 8.82 ± 0.05, using initial concentrations of H_2_O_2_ and CuL of 1.17 × 10^–2^ mol/L and 2.67 × 10^–4^ mol/L, respectively.^21^

Immobilizing complexes on the surface of polymeric membranes enables the development of heterogeneous Cu-Fenton-like systems, which combine the catalytic degradation of organic pollutants with the physical retention of contaminants and byproducts, reducing the need for additional catalyst separation steps.^23,24^ Additionally, the generation of reactive radicals in Cu-Fenton-type reactions can endow membranes with self-cleaning properties, minimizing the fouling problems that are typical of membrane systems.^25–28^

Despite reported advances in catalytic membranes,^26,29,30^ studies describing copper-based catalytic membranes operating in filtration systems under environmentally relevant conditions remain relatively limited, particularly those simultaneously addressing near-neutral pH, operation in the absence of light, catalytic performance, and storage stability. Catalytic membranes are still underexplored for the treatment of wastewater from the petroleum industry and have been more commonly investigated for the degradation of dyes and pharmaceuticals in agitated systems under visible or ultraviolet (UV) irradiation.^31^ In this context, the present study advances the field by developing a PDA-modified nylon membrane decorated with a copper­(II) complex (CuL), which integrates adsorption, catalytic oxidation, and membrane separation into a single material. The proposed system was evaluated in batch mode for the removal of DRX-6BN and in filtration mode for the treatment of an oil-in-water emulsion under mild operating conditions, with additional assessment of storage stability.

## 2 Materials and Methods

### 2.1 Materials and Reagents

Salicylaldehyde (C_7_H_6_O_2_, 98.0%), ethanolamine (C_2_H_7_NO, ≥99.0%), tris­(hydroxymethyl)­aminomethane hydrochloride (Tris–HCl, C_4_H_11_NO_3_·HCl, ≥99.0%), and dopamine hydrochloride (DA, C_8_H_11_NO_2_·HCl) were purchased from Sigma-Aldrich (Rio de Janeiro, RJ, Brazil). Methanol (MeOH, CH_3_OH, ≥99.8%) and copper­(II) acetate monohydrate (Cu­(OAc)_2_·H_2_O, ≥98.0%) were supplied by Vetec (Rio de Janeiro, RJ, Brazil). Commercial neutral nylon membranes (membrane roll, 300 mm × 3000 mm, 0.45 μm pore size) were acquired from GVS North America (Sanford, ME, USA). Hydrogen peroxide (H_2_O_2_, 50% v/v), used in the catalytic experiments, was provided by Sumatex Produtos Químicos Ltd.a. (Rio de Janeiro, RJ, Brazil). Drimaren Red X-6BN (DRX-6BN, CI Reactive Red 243), supplied as a powder, was obtained from Clariant (Rio de Janeiro, RJ, Brazil).

Crude oil with an API gravity of 28°, originating from a Brazilian offshore production unit, was emulsified to generate a synthetic oil-in-water (o/w) emulsion used as a model oily wastewater with characteristics representative of dispersed oil systems Crude oil with an API gravity of 28°, originating from a Brazilian offshore production unit, was emulsified to generate an oil-in-water (o/w) emulsion used as a model oily wastewater for evaluating the performance of the membranes. Emulsification was performed using a high-shear homogenizer (Ultra-Turrax T-25, IKA Works, Staufen, Germany) operating at 15,000 rpm and 60 °C, following a procedure adapted from the literature.^13,32^ All reagents were used without prior purification, and deionized water was used to prepare all the solutions and emulsions.

### 2.2 Synthesis of the Cu­(II) Complex, Membrane Modification, and Characterization

CuL was synthesized in situ according to the methodology reported by Silva et al.^21^ The Schiff base ligand was obtained via an equimolar condensation reaction between salicylaldehyde and ethanolamine in MeOH under magnetic stirring for 120 min. Subsequently, a methanolic solution of Cu­(OAc)_2_·H_2_O was added to the ligand solution, producing a dark green mixture that was stirred for 120 min. CuL was then separated via filtration. The molar ratio of Cu­(OAc)_2_·H_2_O to the ligand was maintained at 1:2. Both the ligand and CuL were synthesized at room temperature (approximately 25 °C). The structural and spectroscopic characterizations of CuL have been reported previously.^21,33^

The nylon membranes were modified following the procedure proposed by Sun et al.,^34^ with minor adaptations. Initially, the membranes were immersed in ethanol for 60 min to remove surface contaminants and processing additives, followed by thorough rinsing with distilled water to remove residual ethanol. A Tris–HCl buffer solution (40 mmol/L, pH 8.5) was then prepared, and the deposition medium was obtained by dissolving 0.2 g of dopamine hydrochloride (DA) in 40 mL of the buffer solution in 250 mL Erlenmeyer flasks under stirring at 160 rpm for 60 min. After this period, PDA was formed via the oxidative self-polymerization of DA. The nylon membranes were then immersed in a PDA-containing solution for an additional 60 min, resulting in nylon/PDA membranes.

Different amounts of CuL (*m*_CuL_) were subsequently introduced into the system, and the nylon/PDA membranes were stirred for 30 min to promote the interaction between the complex and the PDA layer. The CuL fractions were then filtered onto the membrane surfaces, followed by the filtration of 5 mL of glutaraldehyde solution to promote cross-linking. The modified membranes were designated as nylon/PDA/CuL-4 (2.89 g/m^2^), nylon/PDA/CuL-20 (14.46 g/m^2^), and nylon/PDA/CuL-40 (28.92 g/m^2^), according to the ratio of m_CuL_ to the effective membrane area. After modification, the membranes were washed with distilled water and dried at room temperature. The dried membranes were stored in sterile polystyrene Petri dishes (60 mm × 15 mm), sealed with Parafilm, and kept in a dry, dark cabinet protected from heat and UV radiation until use.

The membranes were characterized by Fourier-transform infrared spectroscopy (FT-IR) using a Shimadzu IRSpirit spectrophotometer (Kyoto, Japan) in the range of 4000–400 cm^–1^. The surface morphology and elemental composition were examined by scanning electron microscopy coupled with energy-dispersive X-ray spectroscopy (SEM–EDS) using an FEI TESCAN VEGA 3 instrument (Brno, Czech Republic) after sputter-coating the samples with a thin layer of gold. Water contact angle (WCA) measurements were performed using a Phoenix-i contact angle analyzer. Hydraulic permeance (HP) was determined in a recirculating filtration setup under transmembrane pressures between 0.5 and 2.5 bar, after a compaction period of 120 min.

FT-IR analysis was employed to confirm the membrane surface modification with PDA and the subsequent incorporation of CuL, using the membrane with the highest CuL loading to minimize the spectral overlap. SEM–EDS analyses were performed on membranes with the lowest and highest CuL contents to assess the surface coverage and copper distribution. The wettability and transport properties were evaluated using the WCA and HP measurements, respectively.

### 2.3 Batch Experiments

Batch experiments were conducted in 250 mL Erlenmeyer flasks containing 200 mL of DRX-6BN solution with an initial concentration (*C*_0_) of 20 mg/L. The assays were performed at the natural pH of the solution (∼6.0), 25 °C, and under constant agitation at 200 rpm. No buffer was used, and the pH was monitored throughout the experiments, remaining at approximately 6.0 in all cases. After transferring the DRX-6BN 20 mg/L solution to the flasks, the membranes were added, the vessels were sealed, and the experiments were initiated. For the catalytic tests, the same procedure was followed with the addition of H_2_O_2_ at a concentration of 20 mg/L after membrane insertion in the batch assays. The concentration of DRX-6BN was monitored as a function of time. The experiments were conducted for 300 min.

### 2.4 Filtration Experiments

The membrane performance was further evaluated in a filtration system operated predominantly in the dead-end mode at a transmembrane pressure of 1 bar. The setup consisted of a feed tank (2 L), a permeation cell, a diaphragm pump, and pressure-regulating valves, with a working volume of 1.5 L. For the catalytic tests, the same procedure was followed with the addition of H_2_O_2_ at a concentration of 20 mg/L in the feed tank. Oil and grease (O&G) and chemical oxygen demand (COD) were determined using an initial O&G concentration (*C*_0_) of 100 ± 5 mg/L. The corresponding initial COD and soluble COD values were 267.72 ± 0.53 mg/L and 124.48 ± 0.58 mg/L, respectively. The pH of the feed solution was approximately 6.0, no buffer was used, and the pH was monitored throughout the experiments, remaining close to this value during operation. Filtration experiments were conducted for up to 120 min.

### 2.5 Analytical Methods and Data Analysis

The concentration of DRX-6BN was determined using a Shimadzu UV-1800 UV–Vis spectrophotometer (Kyoto, Japan) at a wavelength of 516 nm. The COD was measured using a Hach DR-2800 spectrophotometer (Loveland, CO, USA) following the standard colorimetric method (5220 D).^35^ O&G concentrations were determined according to the standard extraction method (5520 D), with absorbance readings recorded at 257 and 300 nm using the same UV–Vis spectrophotometer.^35–37^ Residual H_2_O_2_ was quantified according to the methodology described by Nogueira et al. using a Hach DR-2800 spectrophotometer (Loveland, CO, USA) and a colorimetric method.^38^

HP was determined from the permeate flux (*J*) calculated using eq 1, which relates the permeate volume (*V*), effective membrane area (*A*), and filtration time (*t*). The permeance was obtained from the slope of the linear relationship between and the applied transmembrane pressure and expressed in L/(h m^2^ bar).

1 $$J=\frac{V}{A\times t}$$

The removal efficiency (% removal) was calculated using eq 2, where *C*_0_ corresponds to the initial DRX-6BN, O&G, or COD concentration and *C* is the DRX-6BN, O&G, or COD concentration at time.

2 $$\%\mathrm{removal}=\left(1-\frac{C}{C_{0}}\right)\times 100$$

In the membrane filtration experiments, solute rejection (Rej) was determined using eq 3, where *C*_P_ and *C*_A_ are the solute concentrations in the permeate and feed, respectively. The evaluated solutes included COD and O&G.

3 $$\%\mathrm{Rej}=\left(1-\frac{C_{P}}{C_{A}}\right)\times 100$$

The adsorption kinetics were evaluated by calculating the adsorption capacity (q) according to eq 4, as a function of *C*_0_, *C*, adsorbent mass (m), and solution volume (V). The kinetic data were interpreted using the linearized pseudo-first-order (PFO) and pseudo-second-order (PSO) models (eqs 5 and 6, respectively). The Elovich model (eq 7) was also applied to describe chemisorption on the heterogeneous surfaces. Additionally, the intraparticle diffusion model proposed by Weber and Morris (eq 8) was employed to assess the contribution of internal mass transfer resistance, where qt represents the amount of adsorbate at time *t*, *k*_d_ is the intraparticle diffusion rate constant (mg/ (g min^0.5^)), and *B*_L_ is the intercept associated with the boundary layer thickness. The kinetic parameters included the rate constants (*k*_1_ and *k*_2_), equilibrium adsorption capacity (*q*_e_), initial adsorption rate (α), and constants β and *k*_d_, which are related to surface heterogeneity and diffusion, respectively. The half-life (*t*_1/2_) and boundary layer thickness were also determined when applicable. Model fitting, including nonlinear and intraparticle diffusion analyses (Figures S5–S14), was performed using Python 3.10 with NumPy 1.26, SciPy 1.11, and Matplotlib 3.8.

4 $$q=\left(\frac{C_{0}-C_{e}}{m}\right)\times V$$

5 $$\ln\left(q_{e}-q_{t}\right)=\ln\left(q_{e}\right)-k_{1}\times t$$

6 $$\frac{t}{q_{t}}=\frac{1}{k_{2}\times q_{e}^{2}}+\frac{t}{q_{e}}$$

7 $$q_{t}=\frac{1}{\beta }\ln\left(\alpha \times \beta \right)+\frac{1}{\beta }\ln\left(t\right)$$

8 $$q_{t}=k_{d}\times t^{1/2}+B_{L}$$

For the experiments conducted in the presence of H_2_O_2_, the kinetic behavior was evaluated using pseudo-first-order removal (PFOr) and pseudo-second-order removal (PSOr) models (eqs 9 and 10, respectively). These models relate the temporal evolution of C and to the corresponding rate constants (*k*_1r_ and *k*_2r_), which were treated as apparent rate constants (*k*_ap_) in the context of the combined removal and degradation processes.

9 $$\ln\left(\frac{C}{C_{0}}\right)=k_{1r}\times t$$

10 $$\frac{1}{C}=\frac{1}{C_{0}}+k_{2r}\times t$$

The Cu content of the decorated membranes and the copper concentrations in the permeate and reservoir samples collected during the filtration experiments were determined using inductively coupled plasma mass spectrometry (ICP-MS, ICPMS-2030, Shimadzu, Kyoto, Japan).

## 3 Results and Discussion

### 3.1 Characterization of CuL-Decorated Nylon Membranes

The FT-IR spectrum of CuL (Figure S1) shows the characteristic bands of this complex, in accordance with previous studies.^21,33^ The nylon membrane showed bands at 3299 and 2934 cm^–1^, attributed to N–H and C–H stretching vibrations, respectively, due to the amino (−NH_2_) and methine (−CH−) groups.^39,40^ Stronger peaks were detected at ∼1630 cm^–1^, typically attributed to carbonyl group stretching (–C=O), and at 1534 cm^–1^, due to the –NH– bond and imine stretching (–C=N–).^39,40^

Lower-intensity bands were observed at 2859, 1465, 1195, 942, 680, and 568 cm^–1^, corresponding to symmetrical CH_2_ stretching, –NH^–^ deformation/CH_2_ oscillatory motion, symmetrical CCH curvature/CH_2_ torsion, C–C stretching, C–C curvature, and O=C–N curvature, respectively.^40^ The membrane modification by PDA generated slight shifts and changes in the intensity of the previous peaks, which can be attributed to the interaction of PDA with the surface as it forms a thin layer. However, the functionalization with CuL led to an increase in the intensity of the bands in the CuL spectrum.

The SEM images of the nylon membrane revealed a surface with visible pores and a heterogeneous fibrous structure with well-defined and interconnected pores (Figure S2a,b). Modification with PDA and subsequent CuL deposition resulted in a more homogeneous surface with a slight loss of pore definition (Figure S2c,d). Higher CuL loadings resulted in a more intense coating, accompanied by the formation of localized aggregates on the membrane surface (Figure S2e,f).

The presence of copper was confirmed by EDS analysis (Figure S3 b,d). Increasing the CuL loading led to a significant increase in the copper content on the membrane surface, as evidenced by the higher intensity and broader distribution of the Cu signal in the elemental maps (Figure S3a,c), as well as by the increase in the Cu peak intensity in the corresponding spectra (Figure S3b,d). The distributions of C, O, and N remained essentially unchanged, whereas the atomic percentage of Cu increased by approximately 1 order of magnitude. The estimated copper contents, calculated based on the theoretical Cu fraction in the CuL complex (16.21 wt %), were 0.34 ± 0.003, 1.58 ± 0.002, and 2.93 ± 0.005 wt % for nylon/PDA/CuL-4, nylon/PDA/CuL-20, and nylon/PDA/CuL-40, respectively (Table 1). This progressive increase is consistent with the stronger Cu signal observed in the EDS maps and spectra. The EDS elemental maps indicated that copper was distributed over the membrane surface, suggesting the relatively uniform immobilization of CuL, although localized aggregates became more evident at higher catalyst loadings.

**Table 1:** Estimated CuL and Cu Contents of the Decorated Membranes, Calculated from the Mass of Immobilized Complex and the Theoretical Copper Fraction in CuL (16.21 wt %)

membrane	wt % CuL	wt % Cu
Nylon/PDA/CuL-4	2.09 ± 0.02	0.34 ± 0.003
Nylon/PDA/CuL-20	9.73 ± 0.01	1.58 ± 0.002
Nylon/PDA/CuL-40	18.07 ± 0.03	2.93 ± 0.005

The nylon membrane showed an initial WCA of 24.50 ± 1.20° (Figure S4a). Modification with PDA and subsequent CuL deposition did not significantly alter this value. However, the effect of the modification was more pronounced on the HP (Figure S4b). Overall, the PDA modification led to an initial drop of approximately 30% (from 1492.21 to 1053.38 L/ (h m^2^ bar)), suggesting possible pore-narrowing. However, modification with larger masses of CuL (nylon/PDA/CuL-40) led to permeance values similar to that of the nylon/PDA membrane (959.11 L/ (h m^2^ bar)), suggesting that the progressive increase in load may facilitate water passage, complementing the effects of the base membrane. Thus, even with the slight decrease in hydrophilicity of the nylon/PDA/CuL-40 membrane, the results suggest that the interaction of CuL with PDA may have created additional pores or increased internal wettability, making the surface less prone to fouling.^41,42^

### 3.2 Adsorption of DRX-6BN on CuL-Decorated Nylon Membranes

Membranes were employed in the removal of DRX-6BN in the absence of H_2_O_2_, with the aim of evaluating the membranes’ ability to remove contaminants by adsorption under controlled and uniform mixing conditions, without interference from concentration gradients typical of flow systems. The results are shown in Figure 1.

**Figure 1:**
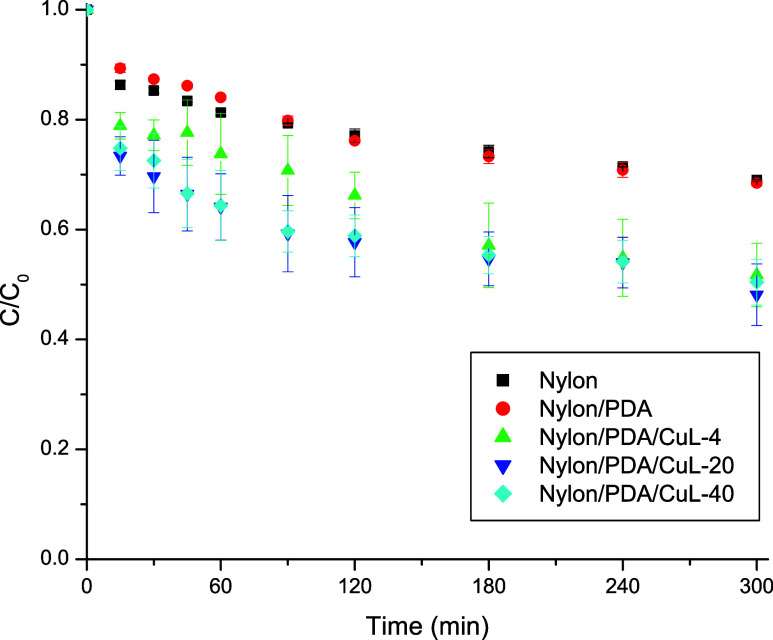
Removal of DRX-6BN in agitated systems using nylon and CuL-decorated membranes. Conditions: pH = ∼6.0, *C*_DRX‑6BN0_ = 20 mg/L, *V*_solution_ = 200 mL, *T* = 25 °C, stirring speed = 200 rpm.

As shown in Figure 1, the unmodified nylon membrane, used as the control, removed approximately 30% of DRX-6BN after 300 min. A similar adsorption performance was observed for the nylon/PDA membrane, indicating that PDA deposition alone did not significantly enhance dye removal under the investigated conditions. In contrast, the incorporation of CuL increased the removal efficiency to approximately 50%, demonstrating the contribution of the immobilized copper complex to pollutant removal. DRX-6BN is a reactive dye containing sulfonate groups, aromatic rings, and several electron-donating functional groups (–N=N–, −OH, and –NH_2_).^21,43^ The increase in removal may be related to the electrostatic attraction between the sulfonated groups of the dye and positive metal centers.

Kinetic adsorption models were used to evaluate the observed dynamics. Figure 2 shows the linearization of the data in Figure 1 according to the PFO, PSO, and Elovich models, respectively. Table 2 shows the linear and nonlinear kinetic parameters calculated for the adsorption of DRX-6BN on the nylon-based membrane and on the CuL-decorated membrane. The graphs corresponding to the nonlinear fits of the data are available in the Supporting Information (Figures S5–S9).

**Figure 2:**
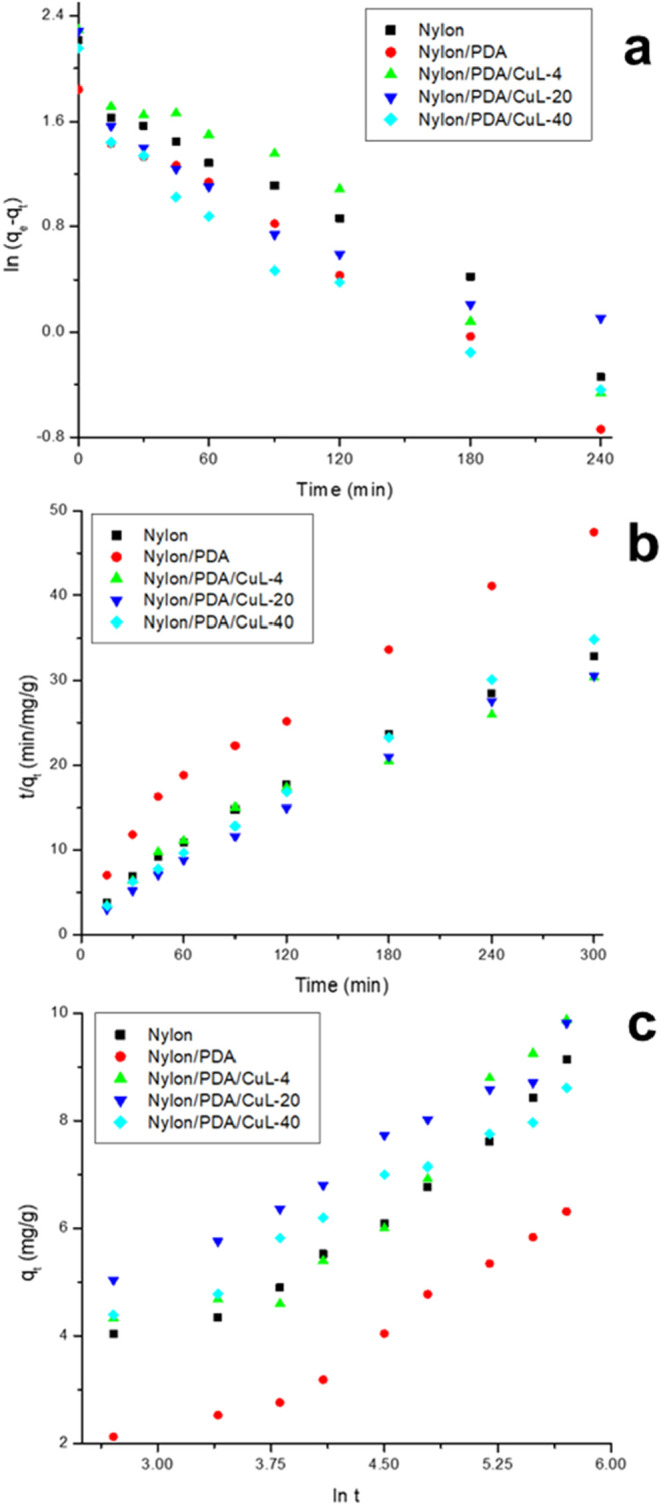
Linearization of adsorption data by the kinetic adsorption models of PFO (a), PSO (b), and Elovich (c) for nylon-based membranes.

**Table 2:** Linear and Nonlinear Kinetic Parameters Calculated for the Adsorption of DRX-6BN on CuL-Decorated Nylon Membranes

	nylon		Nylon/PDA		Nylon/PDA/CuL-4		Nylon/PDA/CuL-20		Nylon/PDA/CuL-40	
model	*R* ^2^	RMSE	*R* ^2^	RMSE	*R* ^2^	RMSE	*R* ^2^	RMSE	*R* ^2^	RMSE
PFO	0.8922	0.8175	0.9422	0.4437	0.7815	1.2984	0.9131	0.7729	0.9389	0.5859
Linear PFO	0.9654	2.4084	0.9902	0.8919	0.9629	1.7168	0.8554	4.3146	0.9076	3.5554
PSO	0.9436	0.5915	0.9668	0.3359	0.8750	0.9820	0.9693	0.4592	0.9801	0.3345
Linear PSO	0.9840	0.5878	0.9822	0.3057	0.9691	0.8020	0.9918	0.5045	0.9961	0.3692
Elovich	0.9803	0.3494	0.9819	0.2483	0.9391	0.6857	0.9936	0.2097	0.9943	0.1786
Linear Elovich	0.9512	0.3440	0.9544	0.3064	0.8896	0.6221	0.9777	0.2047	0.9806	0.1803

As shown in Figures 2 and S5–S9 and in Table 2, all membranes, except for the nylon/PDA membrane, which showed a better fit to the linear PFO model, showed better fits to the linear PSO and nonlinear Elovich models. However, it is important to emphasize that the *R* ^2^ values for nylon/PDA were high for the linear PSO and Elovich models (*R* ^2^ > 0.9819). The lowest RMSE values were observed for the Elovich model. The good fit of the data to the PSO and Elovich models indicates that chemisorption predominated.^44,45^

The best fit by the Elovich model suggests that the surface of the decorated membranes is heterogeneous, with different types of active sites and adsorption energies, reflecting the complexity of the CuL-containing membrane. The visible coloration of the membrane after adsorption reinforces the occurrence of strong chemical interactions between DRX-6BN and the active sites on the membrane surface, which indicates chemisorption, as suggested by kinetic models, especially the Elovich model, which also indicates a heterogeneous surface with different adsorption affinities.

The progressive modification of nylon membranes with PDA and CuL resulted in significant changes in the kinetic parameters described by the nonlinear Elovich model. The base nylon membrane exhibited α = 0.4577 and β = 0.4750, indicating a moderate initial adsorption rate and a relatively homogeneous surface. With the introduction of PDA, a significant reduction in the initial rate (α = 0.1627) and a slight increase in β = 0.5015 were observed, suggesting that the polymeric layer decreased the immediate accessibility to the active sites, although it promoted slightly stronger and more stable interactions.

The modification with the lower mass of CuL increased α to 0.8877 and β to 0.5623, indicating the emergence of more reactive metallic sites and a greater surface heterogeneity. The nylon/PDA/CuL-20 membrane exhibited the highest α (2.6117) and β (0.6702) values, indicating a high initial reactivity associated with the abundant copper active sites. However, with increasing CuL loading, α decreased to 1.7326, accompanied by a slight increase in β to 0.6945. This behavior may be related to surface saturation or partial pore blockage by CuL.

The intraparticle diffusion model (Figures S10–S14) indicates that adsorption can be divided into two stages for all materials. The intercept provides the value of the constant associated with diffusion resistance. Because this value is greater than zero for all samples, the initial stage can be attributed to diffusion in the film (the boundary layer). Thus, it is possible to suggest that the energy disseminated on the surface of the membranes was irregular and that the mechanism involved chemisorption, with film diffusion in the first stage and intraparticle diffusion in the later stage for all the membranes analyzed.

### 3.3 Catalytic Activity of CuL-Decorated Nylon Membranes

The catalytic activity of the CuL-decorated membranes was evaluated in agitated systems in the presence of H_2_O_2_. Figure 3 shows the removal of DRX-6BN, H_2_O_2_ consumption, and adjustments to the PFOr and PSOr kinetic models for the CuL-decorated nylon membranes. Table 3 presents the kinetic parameters values. As reported in a previous study, H_2_O_2_ alone is not efficient for DRX-6BN removal.^21^

**Figure 3:**
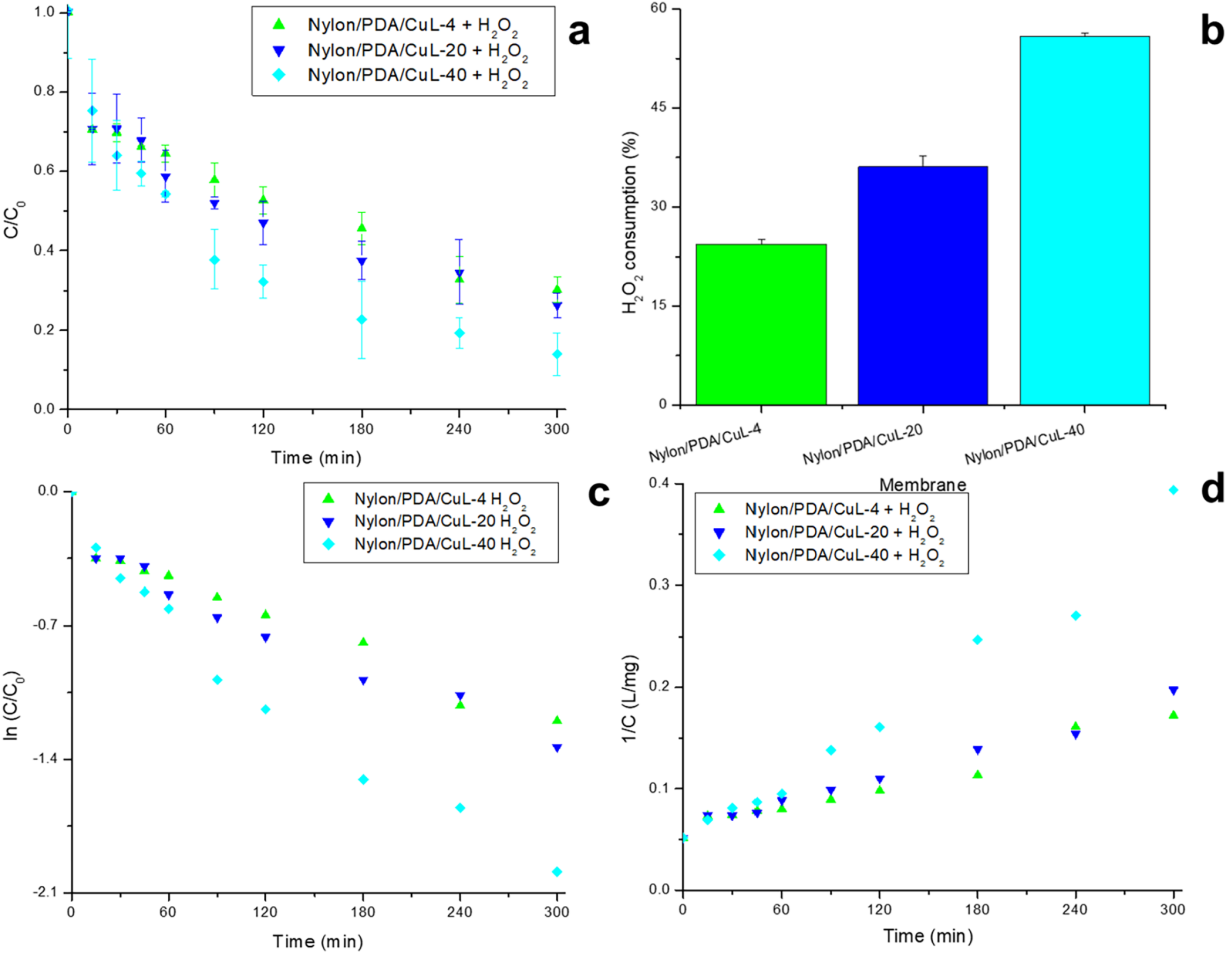
Removal of DRX-6BN (a), and consumption of H_2_O_2_ in 300 min in stirred systems (b), linearization of kinetic data by PFOr (c), and PSOr (d) using CuL-decorated nylon membranes. Conditions: pH = ∼6.0, *C*_DRX‑6BN0_ = *C*_H2O20_ = 20 mg/L, *V*_solution_ = 200 mL, *T* = 25 °C, stirring speed = 200 rpm.

**Table 3:** Kinetic Parameters for Fitting Data to DRX-6BN Removal Kinetics Models on CuL-Decorated Nylon Membrane

	Nylon/PDA/CuL-4		Nylon/PDA/CuL-20		Nylon/PDA/CuL-40	
model	*k*_ap_	*R* ^2^	*k*_ap_	*R* ^2^	*k*_ap_	*R* ^2^
PFOr	3.5 × 10^–3^ min^–1^	0.9426	3.8 × 10^–3^ min^–1^	0.9400	6.3 × 10^–3^ min^–1^	0.9585
PSOr	4.0 × 10^–4^ L/(mg min)	0.9631	4.0 × 10^–4^ L/(mg min)	0.9836	1.1 × 10^–3^ L/(mg min)	0.9790

Membranes containing CuL were efficient in removing DRX-6BN, showing continuous removal over time for both cases, as shown in Figure 3a. Removal was greater in systems with a higher CuL load, reaching more than 80% in 180 min in both cases in the presence of H_2_O_2_, and 86.32 ± 3.66% with the nylon/PDA/CuL-40 membrane in 300 min.

Materials containing metals such as iron, copper, cobalt, or nickel can catalyze the decomposition of H_2_O_2_ into highly reactive hydroxyl radicals (HO^•^), which can promote the mineralization of organic compounds in Fenton reactions and their variations.^21,46,47^ The catalytic activity of CuL in Fenton-like reactions was previously demonstrated by Silva et al.^21^

The gradual increase in the removal of organic compounds as the metal load increases is known.^48^ This is generally attributed to the provision of a greater number of reactive sites, which may enhance H_2_O_2_ activation and the generation of reactive oxidizing species under suitable conditions.^49,50^

The evaluation of H_2_O_2_ consumption (Figure 3b) indicates that the oxidant consumption was incomplete. For the membrane with the highest loading, the consumption was approximately 56%, whereas, for the membrane with the lowest loading, it was below 25%. The concentration of H_2_O_2_ is an important variable in Fenton reaction-based systems and their variations. Complete consumption before the treatment is finished compromises the process efficiency. The sequestration of HO^•^ is known; however, it is in excess.^51–54^

Regarding the kinetic study (Figure 3c,d and Table 3), it is possible to observe that both the PFOr and PSOr models presented satisfactory *R* ^2^ values. However, the PSOr model best fit the results. In any case, a comparison between the *k*_ap_ constants shows that their values increased as CuL loading on the membranes increased.

CuL has been previously studied in solution for the removal of DRX-6BN. Fitting the data to PFOr kinetics resulted in a *k*_ap_ of 0.0152 min^–1^, using a 20 mg/L solution, an initial H_2_O_2_ concentration of 0.0117 mol/L (∼400 mg/L), and a CuL concentration of 0.000533 mol/L (∼42 mg) under the same conditions, promoting the removal of 69.14 ± 1.68% in 60 min.^21^ This value of *k*_ap_ was approximately three times higher than that obtained with membranes containing an equivalent mass of CuL. However, the H_2_O_2_ concentration used in this study was 20 times higher. Although some of the removal was due to adsorption, the results were promising.

The electrochemical behavior of CuL has been previously investigated by cyclic voltammetry, confirming the presence of well-defined anodic and cathodic peaks associated with the Cu^+^/Cu^2+^ redox pair.^33^ This redox activity is consistent with the catalytic role of CuL in the decomposition of H_2_O_2_, potentially leading to the generation of highly reactive HO^•^ species.^21^ In the combined adsorption-oxidation system employing nylon/PDA/CuL in the presence of H_2_O_2_, pollutants (P) initially concentrated on the membrane surface are likely removed through coupled adsorption and Fenton-like oxidation processes occurring at or near the CuL catalytic sites (eqs 11–15), as reported in previous studies.^13,21,55,56^ However, the specific reactive species involved were not directly identified in this study. Therefore, the proposed equations are intended to represent a plausible Fenton-like pathway rather than a definitive mechanistic scheme.

11 $$P_{\mathrm{solution}}+\sup\to P_{\sup}$$

12 $${\mathrm{Cu}}^{\mathrm{II}}\text{−}L+H_{2}O_{2}\to {\mathrm{Cu}}^{I}\text{−}L+{{\mathrm{HO}}_{2}}^{•}+H^{+}$$

13 $${\mathrm{Cu}}^{I}\text{−}L+H_{2}O_{2}\to {\mathrm{Cu}}^{\mathrm{II}}\text{−}L+{\mathrm{HO}}^{•}+{\mathrm{OH}}^{-}$$

14 $${\mathrm{Cu}}^{\mathrm{II}}\text{−}L+{{\mathrm{HO}}_{2}}^{•}\to {\mathrm{Cu}}^{I}\text{−}L+O_{2}+H^{+}$$

15 $$P_{\sup/\mathrm{solution}}+{\mathrm{HO}}^{•}\to \mathrm{oxidized}\;\mathrm{products}$$

The comparison between the adsorption-only and H_2_O_2_-assisted systems indicated that adsorption accounted for a substantial fraction of DRX-6BN removal, whereas the additional removal observed in the presence of H_2_O_2_ reflected the contribution of catalytic oxidation. For the membrane with the highest CuL loading, the removal increased from approximately 50% in the absence of H_2_O_2_ to values above 80% when the oxidant was added, demonstrating the beneficial effect of oxidant addition.

Additional mechanistic insights were obtained through scavenging experiments using MeOH, a commonly used HO• scavenger. As shown in Figure 4, the presence of MeOH significantly suppressed DRX-6BN removal in the adsorption-oxidation system, leading to behavior similar to that observed for adsorption alone. This finding suggests that radical species, particularly HO^•^, play an important role in the catalytic reactions. However, the persistence of partial removal indicates that adsorption and/or nonradical pathways, including the possible involvement of high-valent copper species, may also contribute to contaminant removal. Therefore, the proposed mechanism should be regarded as a plausible pathway supported by indirect scavenging evidence rather than as a fully established exclusive radical route.

**Figure 4:**
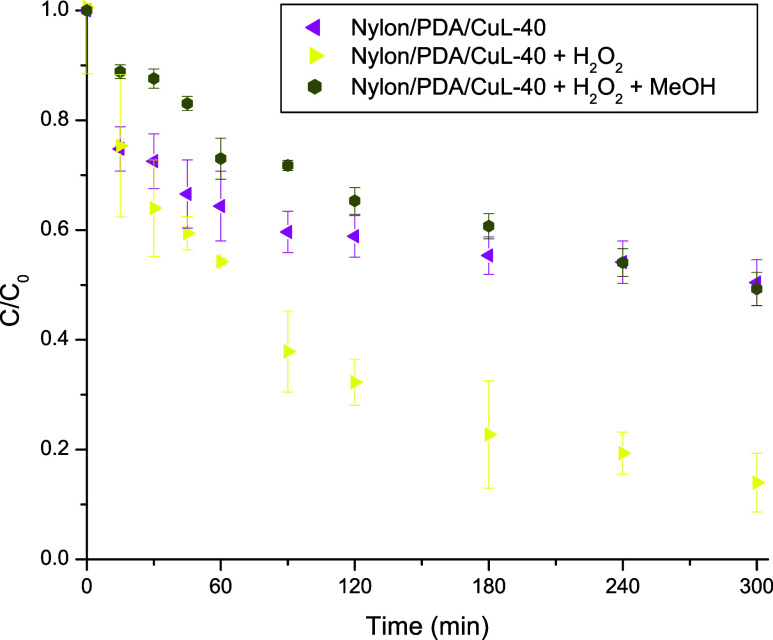
Comparison of DRX-6BN removal by Nylon/PDA/CuL-40 membrane in the absence and presence of H_2_O_2_ and H_2_O_2_ and MeOH in agitated systems. Conditions: pH = ∼6.0, *C*_DRX‑6BN0_, *C*_H2O20_ = 20 mg/L (in oxidation tests and MeOH effect), [MeOH] = 10 × 10^–4^ mol/L (in MeOH effect tests), *V*_solution_ = 200 mL, *T* = 25 °C, stirring speed = 200 rpm.

In agitated systems, although a contribution from homogeneous catalysis cannot be excluded, the overall behavior is consistent with a process likely dominated by surface-mediated pathways, in agreement with the adsorption behavior and diffusion characteristics discussed (Figures 1 and S5–S14).

### 3.4 Performance of CuL-Decorated Nylon Membranes in the Treatment of Oily Wastewater

The experiments performed with DRX-6BN were used as a comparative basis for selecting the membrane to be evaluated for oily wastewater treatment. As shown in Figure 1, the CuL-decorated membranes presented similar adsorption capacities, indicating that increasing the CuL load did not lead to substantial gains in pollutant removal under the conditions investigated. Therefore, the nylon/PDA/CuL-4 membrane, which contained the lowest CuL load, was selected for the filtration experiments. This choice minimized catalyst consumption and the potential release of copper while maintaining satisfactory removal performance, thus representing a more economical and environmentally attractive configuration. Figure 5 shows the results obtained for O&G rejection and COD removal with time.

**Figure 5:**
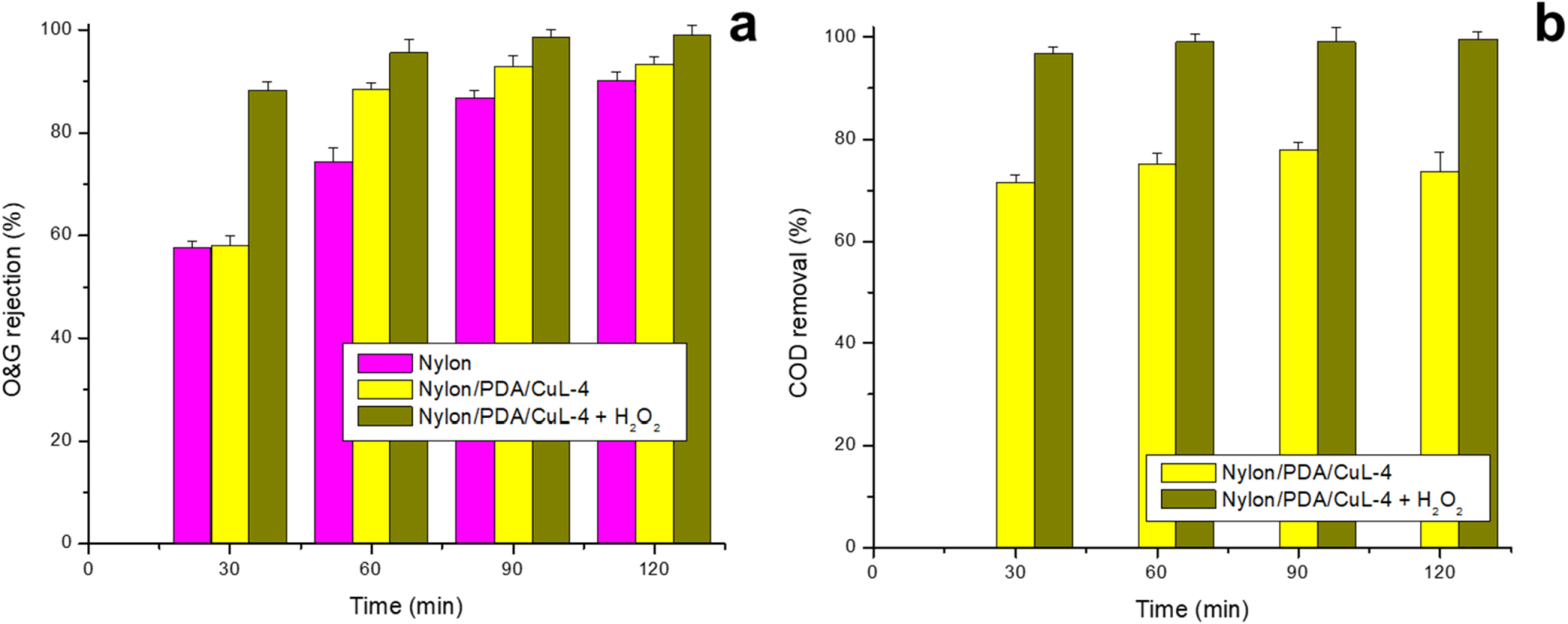
O&G rejection (a) and COD removal (b) from oily wastewater using CuL-decorated nylon membranes in filtration system. Conditions: pH = ∼6.0, *C*_O&G0_ = 100 ± 5 mg/L, *C*_H2O20_ = 20 mg/L (in oxidation tests), *V*_emulsion_ = 1.5 L, *T* = 25 °C, and *P* = 1 bar.

As shown in Figure 5a, O&G rejection increased over time, reaching values of 90.01 ± 1.75%, 93.19 ± 1.57%, and 98.85 ± 2.08% for the base membrane and those containing CuL in the absence and presence of H_2_O_2_ at 120 min of filtration. These results can be attributed to the greater vulnerability of nylon to fouling during oily wastewater treatment.^57^ Regarding COD removal (Figure 5b), the nylon base membrane was removed by 46.66 ± 1.89% in 120 min, whereas the removal of organic compounds was continuous in the systems with modified membranes. The removal was 99.54 ± 1.56% in 120 min in the system with the addition of H_2_O_2_, being greater than 95% at 30 min of filtration.

Halim et al. reported the modification of a nylon membrane with ZIF-8 to increase its mechanical strength and permeability.^58^ This membrane achieved an oil rejection of 89%, which was several times greater than that of the pure nylon membrane. An o/w separation membrane was prepared by adding ZnO nanoparticles to nylon nanofibers, and it exhibited an oil removal rate of greater than 90%.^59^

Comparing the system used to remove COD from oily wastewater in the petroleum industry is difficult because the performance of catalytic membranes with Fenton oxidation and its variations are more commonly studied for the degradation of dyes and pharmaceuticals in agitated systems and in the presence of visible or ultraviolet (UV) light.^31^

The permeate flux results showed that the modified membranes exhibited superior hydraulic performance compared to the pure nylon membrane, especially in the presence of H_2_O_2_ (Figure S15a,b). In Fenton-like systems, the generation of reactive species, particularly HO^•^, can promote the partial degradation of organic compounds adsorbed on the membrane surface, contributing to a self-cleaning effect and mitigating fouling.^60^ This behavior is consistent with the higher flux and normalized flux values observed for the nylon/PDA/CuL-4 membrane in the presence of oxidant. Furthermore, the nylon base membrane already exhibited hydrophilic character, and the modification with 4 mg of CuL did not promote significant changes in the contact angle with water (Figure S4a).

To better understand the fouling mechanisms during oil-in-water emulsion treatment, the permeate flux data over time were fitted to Hermia’s classical models: total pore blockage (TPB), pore obstruction (PO), partial pore blockage (PPB), and filter cake (FC) (Table S1). Figure S16 presents the linear fits, and Table S2 lists the coefficients of determination (*R* ^2^) and fouling constants (*K*) for each model. In general, the TPB and PO models provided the best fits, indicating that fouling was predominantly controlled by intrapore mechanisms associated with the blockage or gradual constriction of pores by oil droplets and adsorbed organic compounds. For the pure nylon membrane, a satisfactory fit to the filter cake model was also observed (*R* ^2^ > 0.90), suggesting an additional contribution from the surface deposition of oil droplets and organic matter on the membrane.

The fouling constant *K* was lower for the nylon/PDA/CuL-4 membrane in the presence of H_2_O_2_, indicating a lower fouling rate. This result suggests that catalytic activation by H_2_O_2_ contributed to reducing the accumulation of organic material on the membrane, favoring greater flux stability over time. This interpretation is consistent with the permeate and normalized flux profiles, which showed better hydraulic performance when catalytic oxidation was coupled with the filtration process.

The progressive increase in O&G rejection over time is also consistent with the formation of a fouling layer on the membrane surface, which may act as a secondary barrier and contribute to the physical retention of the oil droplets. Therefore, the overall performance can be attributed to the synergistic combination of physical separation and catalytic oxidation, in which fouling contributes to pollutant retention, whereas catalytic activity mitigates its development.

While the batch experiments were designed to isolate the effects of adsorption and H_2_O_2_-assisted catalytic oxidation on the removal of DRX-6BN, the filtration experiments evaluated the membrane under continuous operation, where physical separation, adsorption, and catalytic oxidation occurred simultaneously. Although a direct comparison between the two systems is limited by differences in matrix composition and operating mode, the results consistently demonstrate that CuL immobilization enhances pollutant removal and that coupling with H_2_O_2_ improves overall performance under mild conditions.

### 3.5 Storage Stability of CuL-Decorated Nylon Membranes and Evaluation of Copper Leaching

The use of copper-containing catalytic membranes, which are active in Fenton-like reactions, presents a high potential for the oxidation of organic compounds via efficient redox mechanisms.^61,62^ However, investigating the maintenance of the future activity of these membranes after storage, a common scenario in industrial operations, is relevant and necessary to ensure that their commercial adoption is not compromised. Figure 6 presents the storage stability results for the decorated membranes in the presence and absence of H_2_O_2_.

**Figure 6:**
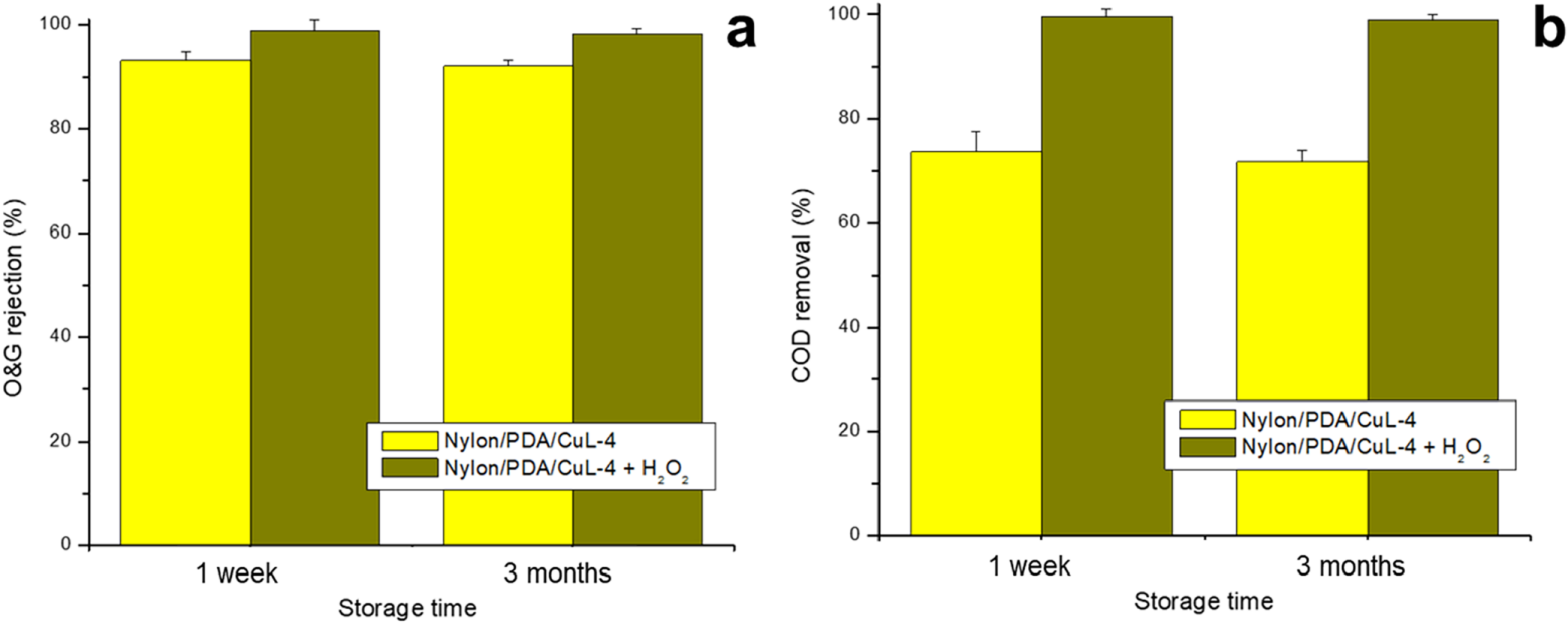
O&G rejection (a) and COD removal (b) of CuL-decorated nylon membranes as a function of storage time. Conditions: pH = ∼6.0, *C*_O&G0_ = 100 ± 5 mg/L, *C*_H2O20_ = 20 mg/L (in oxidation tests), *V*_emulsion_ = 1.5 L, *T* = 25 °C, *P* = 1 bar.

As shown in Figure 6, the removal of both O&G concentration and COD remained practically unchanged over 3 months, both for the tests in which the material was used in filtration systems without H_2_O_2_ and for those in which oxidation was coupled with filtration. These results suggest that the materials preserved their catalytic properties for at least three months under the storage conditions described in Section 2.2.

Studies with longer storage periods are needed for a more complete evaluation of the behavior of membranes in long-term applications for the treatment of oily wastewater. Although the studied interval was short, it allowed for the observation of the initial stability trends.

Although immobilizing the CuL complex on the nylon membrane contributes to increased process efficiency and potentially reduces catalyst release compared to its use in solution, quantifying copper leaching is fundamental for a more comprehensive assessment of the stability and environmental safety of the material. Copper can be toxic to aquatic and terrestrial organisms when released at high concentrations.^63,64^ To further assess the stability of the immobilized CuL complex, additional filtration experiments were conducted using deionized water under the same operating conditions employed in the oily wastewater tests, in the presence and absence of H_2_O_2_. The copper concentrations in the permeate were monitored over time using ICP-MS (Figure 7).

**Figure 7:**
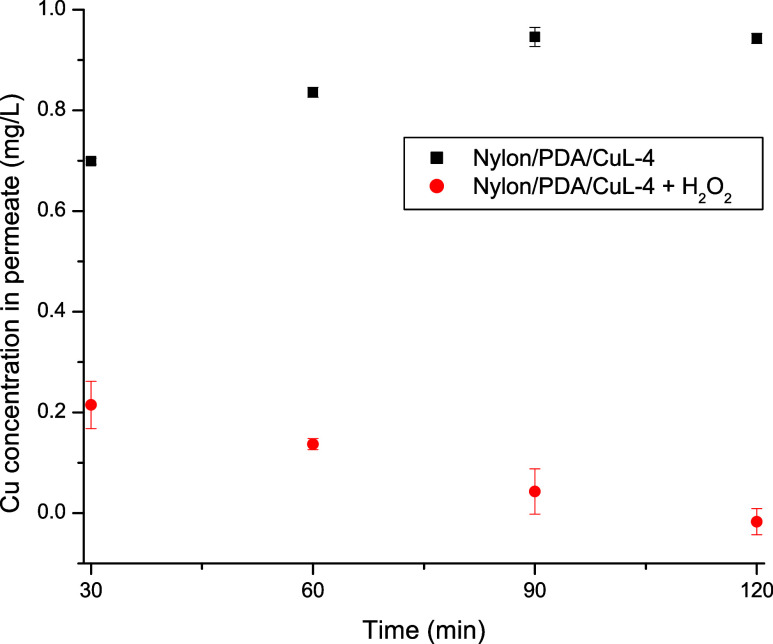
Copper concentration measured by ICP-MS in permeate samples collected during filtration experiments performed in the absence and presence of H_2_O_2_ using deionized water as feed. Conditions: pH = ∼6.0, *C*_H2O20_ = 20 mg/L (in oxidation tests), *V* = 1.5 L, *T* = 25 °C, *P* = 1 bar.

In the absence of H_2_O_2_, the Cu concentrations in the permeate ranged from 0.699 ± 0.006 to 0.946 ± 0.019 mg/L. In the presence of H_2_O_2_, substantially lower values were observed, varying from 0.215 ± 0.047 to concentrations below the analytical detection limit after 120 min of reaction. Additionally, the copper concentration measured in the feed reservoir after 120 min in the H_2_O_2_-assisted leaching system was 0.222 ± 0.042 mg/L, whereas the Cu concentrations remained below the detection limit in the absence of H_2_O_2_.

The maximum theoretical Cu concentration corresponding to the complete release of Cu from the membrane was estimated to be 6.49 mg/L. Therefore, all measured concentrations were markedly lower than this value, suggesting that most of the immobilized copper remained associated with the membrane during operation. These results demonstrate limited copper release and suggest that the CuL complex remained stable under the investigated conditions, even in the presence of H_2_O_2_.

The copper concentrations measured in the permeate remained below 1.0 mg/L under all investigated conditions, with substantially lower values observed in the presence of H_2_O_2_. These concentrations are below the maximum levels or guideline values established by several regulatory agencies for drinking water, including 1.3 mg/L by the U.S. Environmental Protection Agency (EPA), 3.0 mg/L by the World Health Organization (WHO), 1.5 mg/L for groundwater, and 1.0 mg/L for surface waters according to Chinese environmental quality standards, and 1.0 mg/L for effluent discharge established by Brazilian legislation.^65,66^ Although drinking water and effluent standards are not directly equivalent, this comparison indicates that copper release from the developed membranes was limited and remained within the concentration ranges considered acceptable by major international regulatory frameworks.

It is important to note that the initial conclusions obtained regarding the system in the presence and absence of H_2_O_2_ need to be complemented by monitoring the copper concentration in the permeate during tests in the presence of contaminants, characterizing the membrane after use, evaluating possible CuL losses under shear conditions, and conducting reuse tests in multiple cycles to validate the applicability of this technology on a larger scale. Furthermore, economic feasibility studies are necessary to assess the potential for practical implementation of the system.

### 3.6 Performance Comparison of the Developed Nylon Membranes with Reported Membranes

The catalytic activity of copper-containing nylon membranes in Fenton-like reactions for the treatment of oily wastewater has been poorly reported,^26^ particularly for copper coordination compound-based membranes. Table 4 summarizes some studies on copper-containing membranes. In addition to the studies mentioned in Table 4, Liu and Cao modified a carboxymethyl chitosan nylon mesh and a cobalt MOF (ZIF-67) to develop a composite capable of absorbing oil and adsorbing dyes.^67^ Although oil separation was mentioned, performance data were not provided.

**Table 4:** Studies on Copper-Based Membranes for Organic Removal and O&G Separation^a^

membrane	target compound (removal)	conditions	o/w separation	refs
Cu@Cu_2_O film	MB (81.86%)	Target compound at 10 mg/L, without H_2_O_2_, removal after 240 min after equilibrium (40 min), visible light.	Gasoline in water stabilized by Tween-20 (>99.5%)	^68^
copper mesh coated with Bi_2_MoO_6_/Cu_3_(PO_4_)_2_	MB (97.5%)	50 mL of the target compound at 10 mg/L, 50 μL of H_2_O_2_, removal after 65 min after equilibrium (20 min), visible light, agitated systems.	Cyclohexane in water stabilized with CTAB (>98.6%)	^69^
CuWO_4_@Cu_2_O film on copper mesh	MB (96.4%) and RhB (90.2%)	50 mL of the target compound at 0.1 mM, 1 mM Na_2_S_2_O_8_, removal after 120 min after equilibrium (30 min), visible light.	Various (>95,0%)	^70^
Nylon/PDA/Ag	MB (97.3%)	10 mL of target compound at 10 mg/L, 1 mL of H_2_O_2_, removal after 15 min, agitated system.	Various (>99%)	^71^
Nylon/PDA/CuL	DRX-6BN (up to 50.59%)	1.5 L of the target compound at 20 mg/L, 20 mg/L of H_2_O_2_, removal after 120 and 300 min, absence of light, filtration system.	Petroleum in water (93.20% and 98.85% (in the presence of H_2_O_2_))	this work

a MB: Methylene blue, RhB: Rhodamine B, DRX-6BN: Drimaren Red X-6BN.

The results obtained in this study demonstrate competitive performance compared to copper-based catalytic membranes reported in the literature. As summarized in Table 4, several previously described systems have shown high efficiencies in dye degradation and oil/water separation but generally operate under more restrictive conditions, such as the use of visible light radiation.^68–71^ However, for large-scale applications, the widespread use of lamps can drastically increase energy consumption and consequently, costs. Furthermore, in large reactors, light penetration into wastewater to activate the catalysts is often difficult because of the interference from suspended substances.^31^

In contrast, the nylon/PDA/CuL membrane developed in this study was able to promote the removal of up to 50.59% of DRX-6BN in a batch system and achieve O&G rejection and COD removal greater than 93% and 98%, respectively, in a filtration system operating in the absence of light and at a near-neutral pH. In addition, this study evaluated the storage stability of the membrane, an aspect that has rarely been addressed in previous studies. These results support the feasibility of the proposed strategy for developing multifunctional catalytic membranes for treating oily wastewater.

However, although the high removal rates observed indicate the effective transformation of contaminants, Fenton and Fenton-like processes generally involve the formation of partially oxidized organic intermediates before complete mineralization occurs. Thus, the removal of COD and color does not necessarily imply the complete conversion of pollutants into CO_2_ and H_2_O, nor does it ensure a reduction in the toxicity of the treated effluent. In this context, total organic carbon (TOC) analyses and ecotoxicological tests are important for a more comprehensive assessment of the efficiency and environmental safety of these processes.

The membrane preparation procedure involves simple immersion and coating steps performed under mild conditions, suggesting the potential for scale-up. Nevertheless, further studies addressing process optimization, catalyst consumption, and economic feasibility are required to assess the industrial applicability of this method.

From a practical perspective, the proposed nylon/PDA/CuL membrane presents distinct features compared to selected benchmark technologies. In contrast to UV/TiO_2_-based photocatalytic systems, the membrane was evaluated in the absence of external irradiation, which may be advantageous for treating turbid or highly colored wastewater, where light cannot penetrate. Compared with conventional homogeneous Fenton-like processes, the immobilization of the CuL complex on a membrane support offers the additional benefit of catalyst localization, thereby reducing the need for catalyst separation from the treated effluent. Furthermore, unlike conventional commercial ultrafiltration membranes, which mainly act as physical barriers and may concentrate contaminants in the retentate, the proposed material was designed to combine physical separation, adsorption, and H_2_O_2_-assisted catalytic oxidation in a single system. These aspects highlight the conceptual advantage of membranes as an integrated treatment platform, although their practical competitiveness still depends on the long-term stability and operational parameters discussed above.

## 4 Conclusions

In this study, PDA-assisted functionalization was used to immobilize a CuL complex on nylon membranes, yielding multifunctional catalytic membranes capable of integrating adsorption, H_2_O_2_ activation, and membrane separation on a single platform. The proposed system operates under mild conditions, including near-neutral pH and the absence of irradiation, which are relevant features for the development of more practical Fenton-like treatment strategies.

The modified membranes improved contaminant removal in batch systems and enabled simultaneous oil–water separation and organic matter removal during filtration. The results also indicated that the combination of physical separation and catalytic oxidation contributed to pollutant removal, whereas the presence of H_2_O_2_ favored better flux behavior and slower fouling development. In addition, the membranes retained their functional properties after three months of storage, indicating the preliminary stability of the material. Overall, these findings provide evidence that CuL-decorated nylon membranes can serve as an integrated adsorption-oxidation-separation platform for wastewater treatment applications, particularly for oily wastewater.

Future studies should comprehensively assess system stability, environmental safety, and practical feasibility. In this context, it is important to monitor copper release into the permeate and quantify metal leaching, as well as to perform postuse membrane characterization and investigate possible losses of the CuL complex under operating conditions, including shear stresses. Reuse tests over multiple cycles, including the evaluation of catalytic activity retention and permeate flux behavior over time, are also fundamental for determining material durability and resistance to fouling. Additionally, economic feasibility analyses, studies with other recalcitrant contaminants, and tests with real effluents or nonsynthetic matrices are necessary to further validate the technology and define its potential for practical-scale application.

## Supplementary Material



## Notes

FT-IR spectra of CuL and nylon-based membranes; micrographs of nylon, nylon/PDA/CuL-4, and nylon/PDA/CuL-40 membranes; elemental mapping and EDS spectrum of nylon/PDA/CuL-4 and nylon/PDA/CuL-40 membrane; water contact angle and permeance of the nylon-based membranes; fitting of DRX-6BN adsorption data on nylon, nylon/PDA, nylon/PDA/CuL-4, nylon/PDA/CuL-20, and nylon/PDA/CuL-40 membranes to the nonlinear PFO, PSO and Elovich models; fitting of DRX-6BN adsorption data on nylon, nylon/PDA, nylon/PDA/CuL-4, nylon/PDA/CuL-20, and nylon/PDA/CuL-40 membranes to the intraparticle diffusion model; permeate flux and *J*/*J*_0_ for the pristine and CuL decorated nylon membranes during oily wastewater treatment; graphs associated with the Hermia models applied to the treatment data with pristine and CuL decorated nylon membranes: BTP, OP, BPP, and TF; equations corresponding to Hermia’s models; fouling parameters data and *R* ^2^ for the Hermia fouling models of the pristine and CuL decorated nylon membranes (PDF)
