# Supplementary material for: Hybrid Process of Oxidation and Separation of Organic Contaminants Using Copper(II) Complex-Decorated Nylon Membranes under Mild Conditions
Source: ACS Omega. 2026 Jun 25;11(26):39239–51. doi: 10.1021/acsomega.6c03603 (PMC13347661; doi:10.1021/acsomega.6c03603)
Supplement: Supplementary file 1 [file ao6c03603_si_001.pdf]

**Hybrid process of oxidation and separation of organic contaminants using copper(II) complex-decorated nylon membranes under mild conditions**

Felipe P. da Silva [a,b,*], Aline C. F. Pereira [a], Juliana C. Pinheiro [a], Annelise Casellato [c], Cristiano P. Borges [d], Fabiana V. da Fonseca [a]

[a] Escola de Química, Universidade Federal do Rio de Janeiro, Av. Athos da Silveira Ramos 149, Bl. E, Cidade Universitária, Rio de Janeiro 21941-909, RJ, Brazil

[b] Faculdade de Engenharia, Universidade do Estado do Rio de Janeiro, Rua São Francisco Xavier 524, Rio de Janeiro, RJ 20550-900, Brazil

[c] Instituto de Química, Universidade Federal do Rio de Janeiro, Av. Athos da Silveira Ramos 149, Bl. A, Cidade Universitária, Rio de Janeiro 21941-909, RJ, Brazil

[d] Instituto Alberto Luiz Coimbra de Pós-Graduação e Pesquisa de Engenharia (COPPE), Universidade Federal do Rio de Janeiro, Av. Horácio Macedo 2030, Cidade Universitária, Rio de Janeiro 21941-972, RJ, Brazil

*email: felipe.psilva@eng.uerj.br

**Supporting Information**

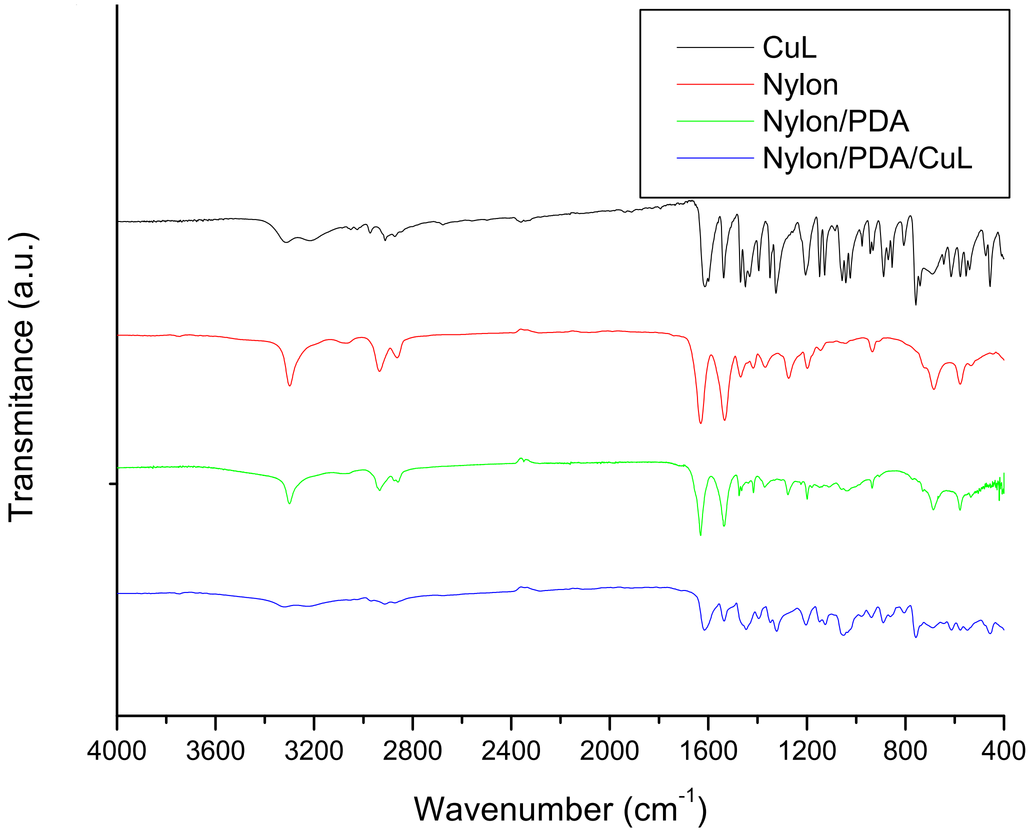


**Figure S1.** FT-IR spectra of CuL and nylon-based membranes

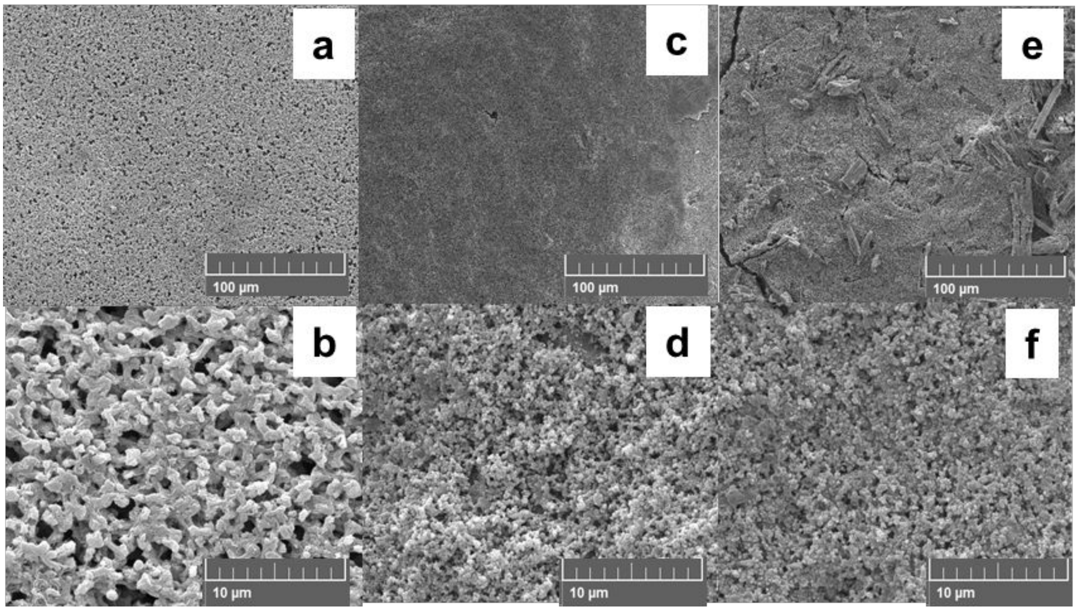

**Figure S2.** Micrographs of nylon 500 x (**a**) nylon 5000 x (**b**), nylon/PDA/CuL-4 500 x (**c**), nylon/PDA/CuL-4 5000 x (**d**), nylon/PDA/CuL-40 500 x (**e**), and nylon/PDA/CuL-40 5000 x (**f**) membranes

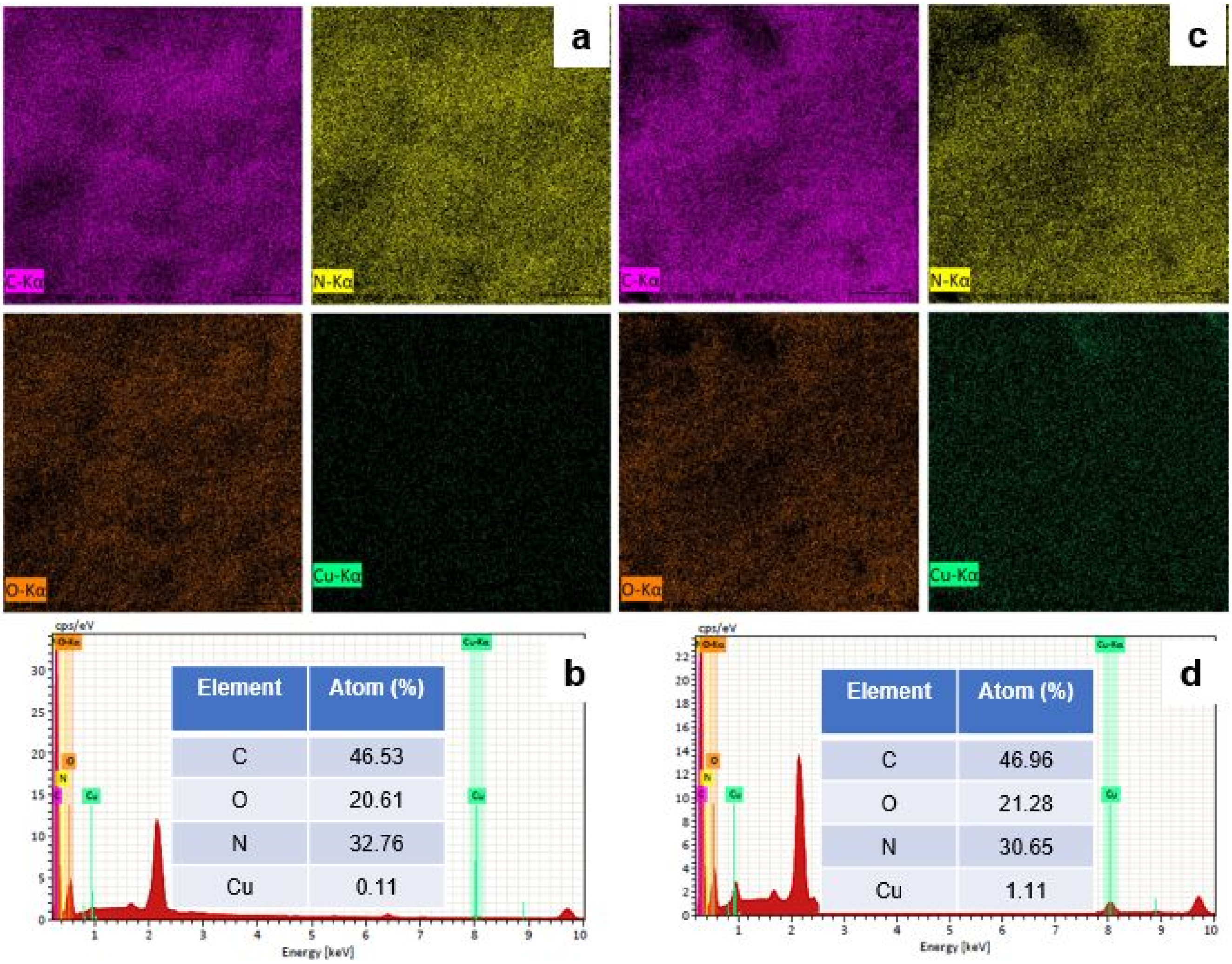

**Figure S3.** Elemental mapping of nylon/PDA/CuL-4 membrane (**a**) EDS spectrum of nylon/PDA/CuL-4 membrane (**b**), Elemental mapping of nylon/PDA/CuL-40 membrane (**c**) EDS spectrum of nylon/PDA/CuL-40 membrane (**d**)

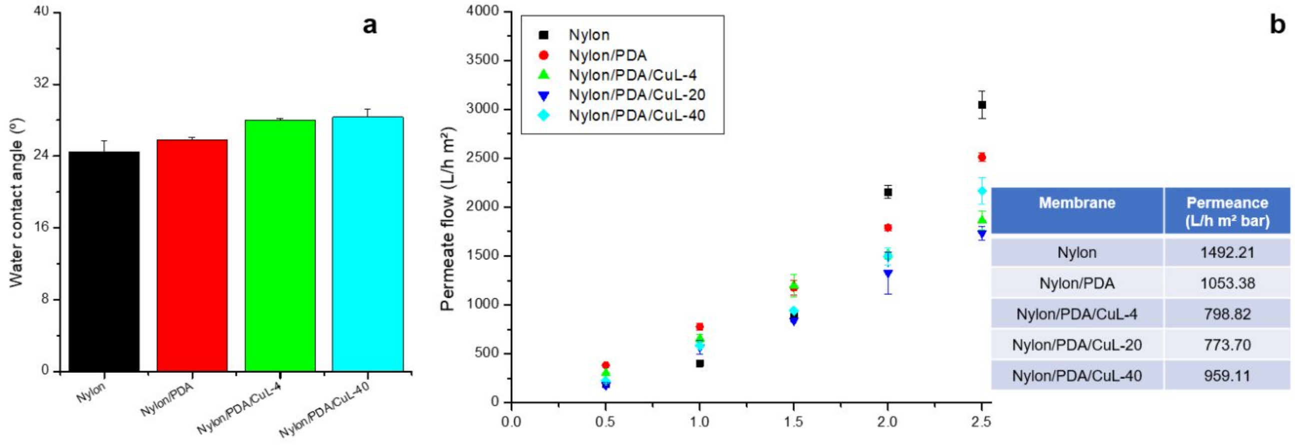


**Figure S4.** Water contact angle (**a**) and permeance (**b**) of the nylon-based membranes

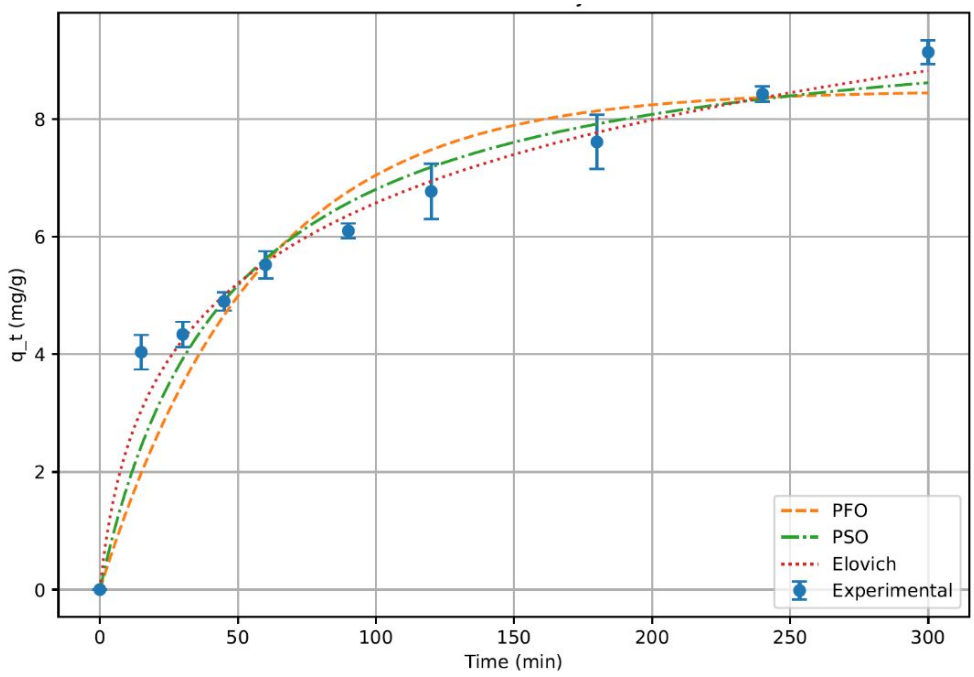


**Figure S5.** Fitting of DRX-6BN adsorption data on nylon membrane to the nonlinear PFO, PSO and Elovich models

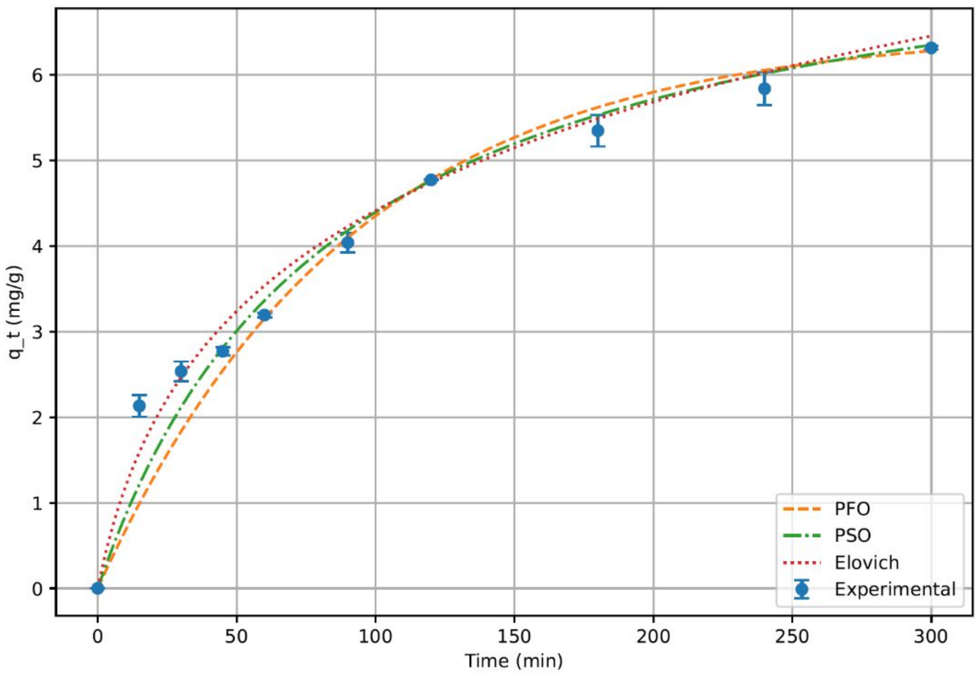


**Figure S6.** Fitting of DRX-6BN adsorption data on nylon/PDA membrane to the nonlinear PFO, PSO and Elovich

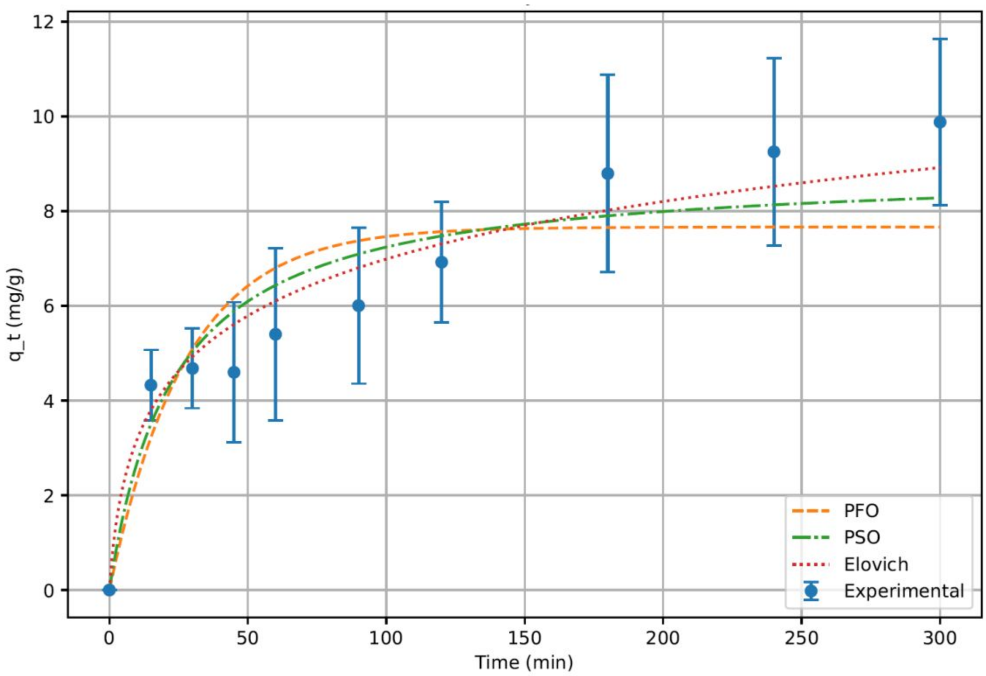


**Figure S7.** Fitting of DRX-6BN adsorption data on nylon/PDA/CuL-4 membrane to the nonlinear PFO, PSO and Elovich models

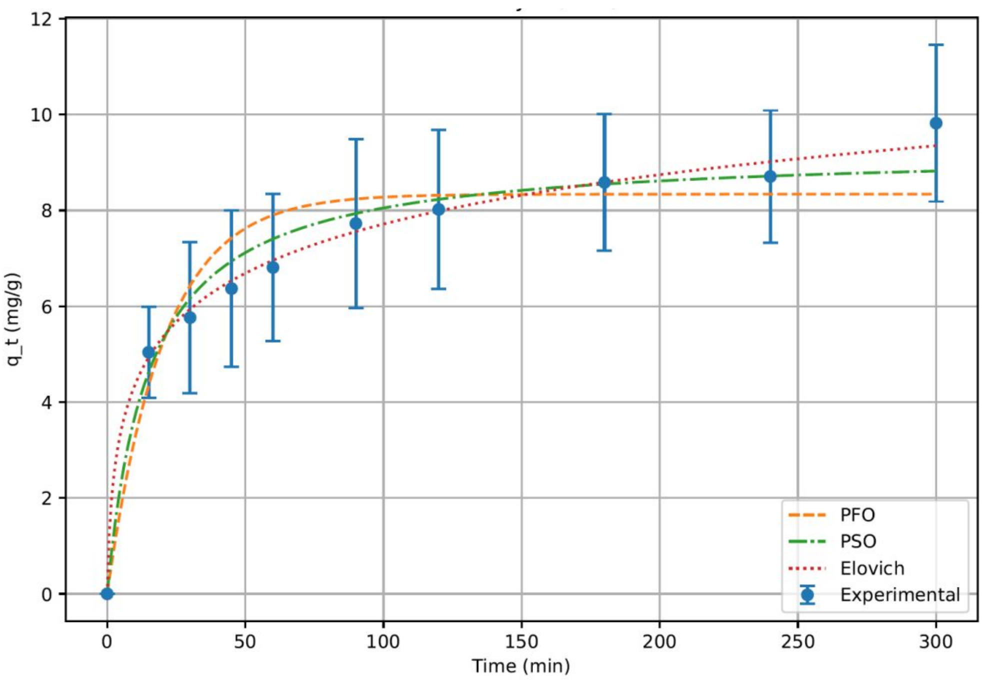


**Figure S8.** Fitting of DRX-6BN adsorption data on nylon/PDA/CuL-20 membrane to the nonlinear PFO, PSO and Elovich

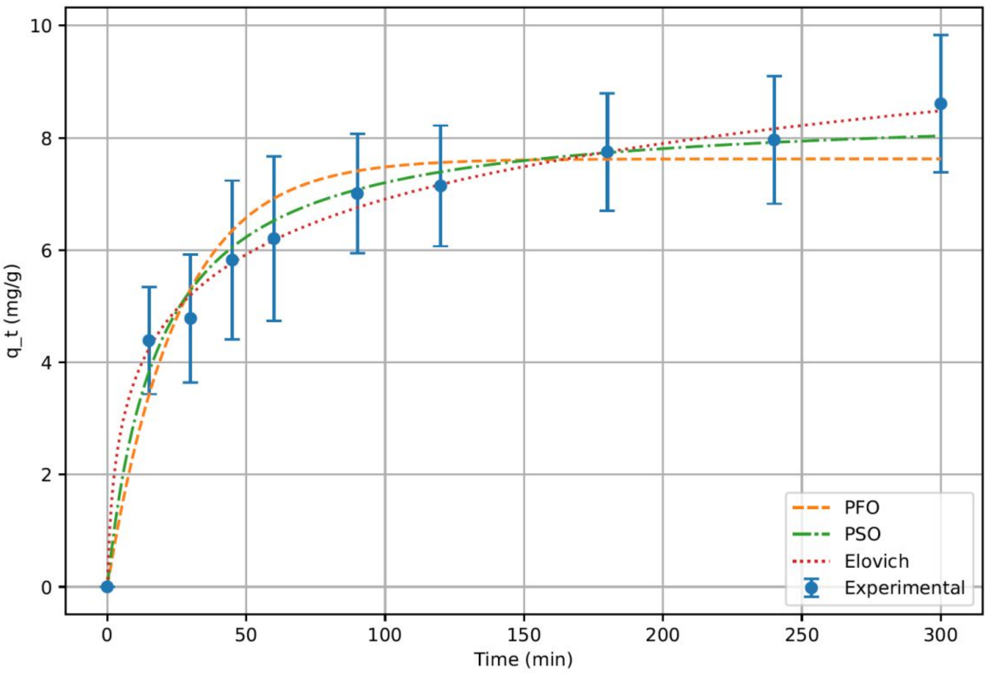


**Figure S9.** Fitting of DRX-6BN adsorption data on nylon/PDA/CuL-40 membrane to the nonlinear PFO, PSO and Elovich models

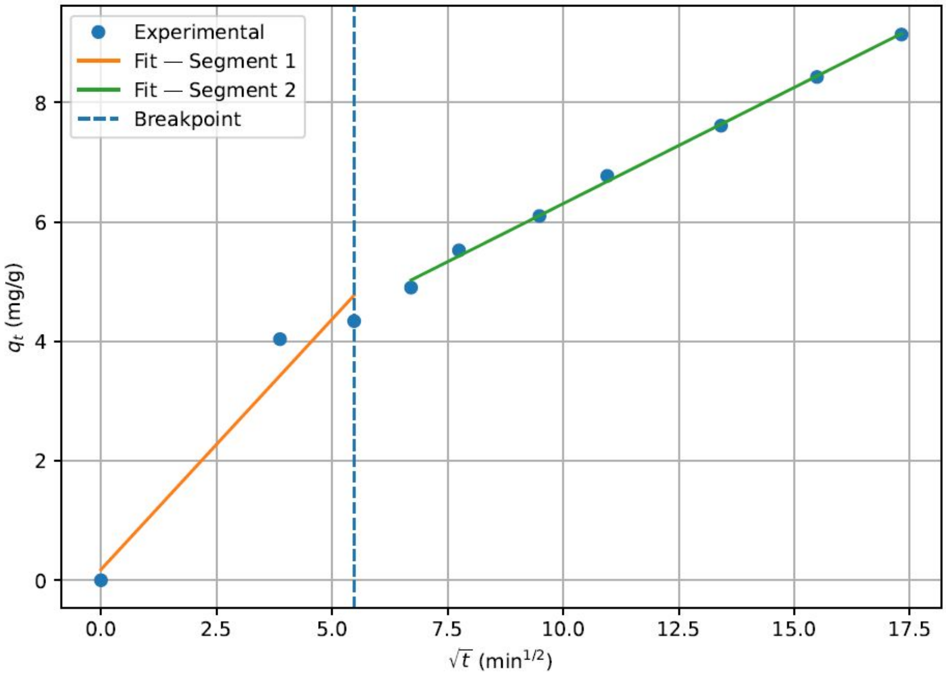


**Figure S10.** Fitting of DRX-6BN adsorption data on nylon membrane to the intraparticle diffusion model

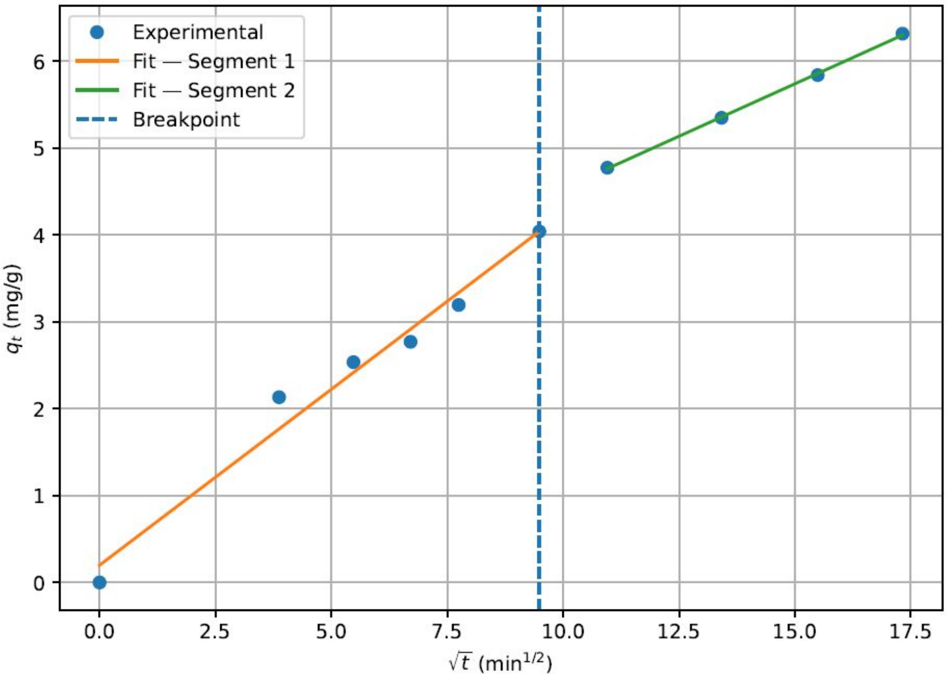


**Figure S11.** Fitting of DRX-6BN adsorption data on nylon/PDA membrane to the intraparticle diffusion model

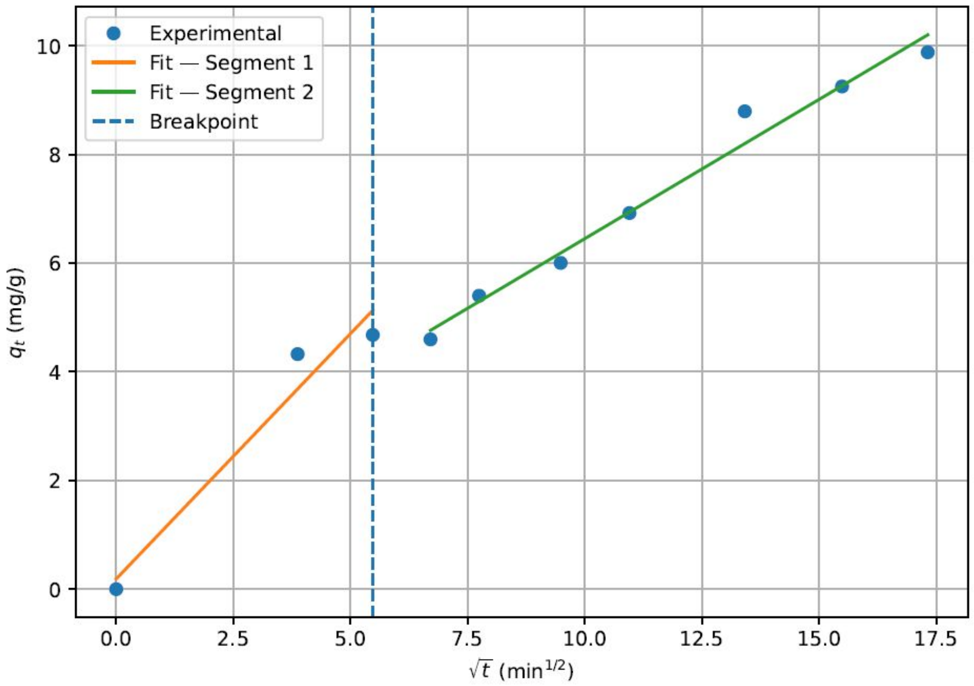


**Figure S12.** Fitting of DRX-6BN adsorption data on nylon/PDA/CuL-4 membrane to the intraparticle diffusion model

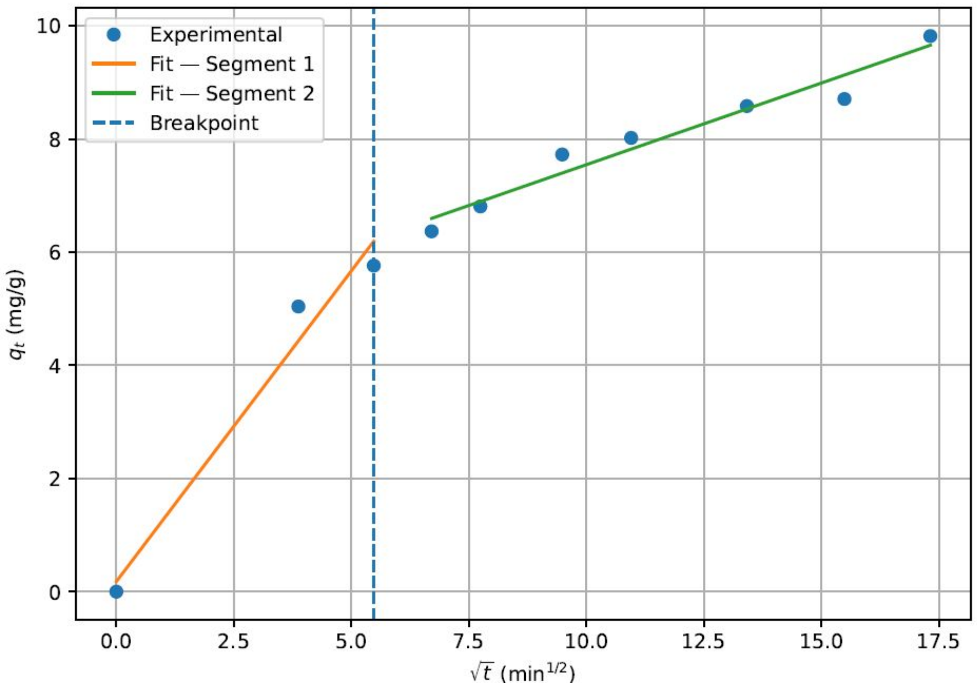


**Figure S13.** Fitting of DRX-6BN adsorption data on nylon/PDA/CuL-20 membrane to the intraparticle diffusion model

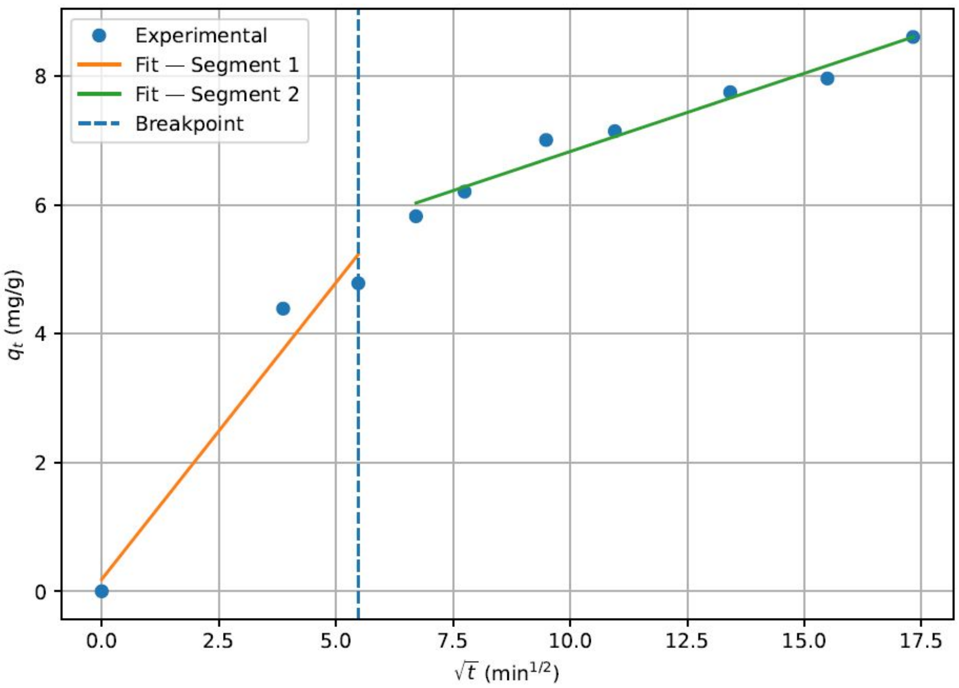


**Figure S14.** Fitting of DRX-6BN adsorption data on nylon/PDA/CuL-40 membrane to the intraparticle diffusion model

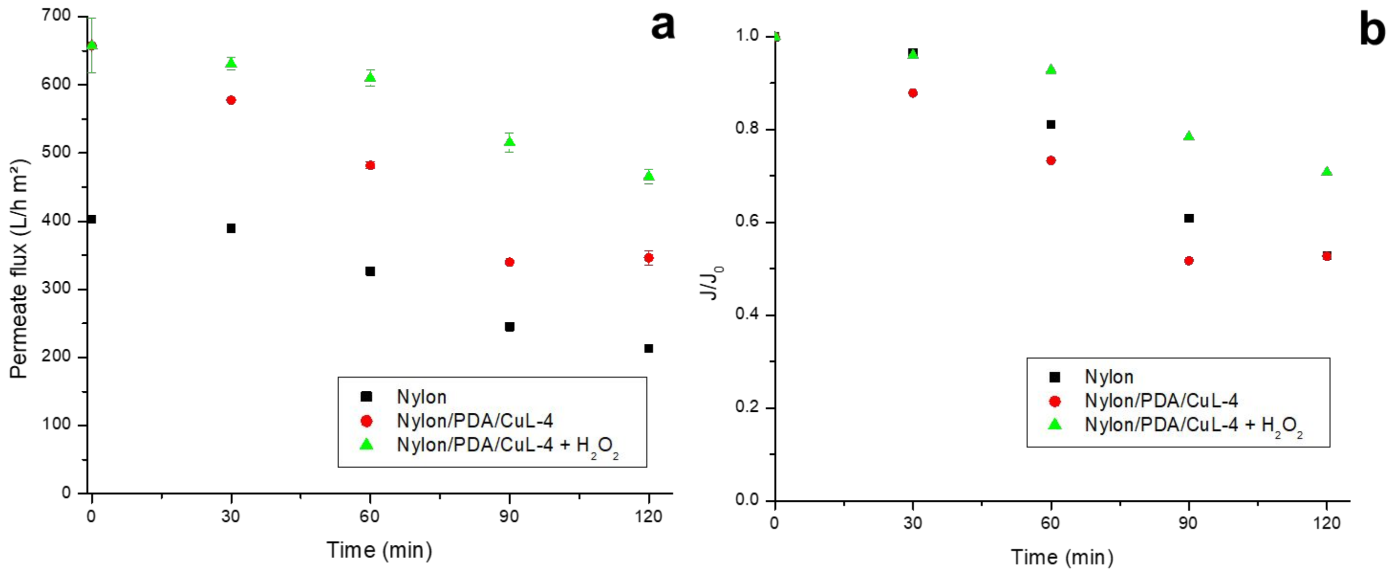


**Figure S15.** Permeate flux (**a**) and J/J0 for the pristine and CuL decorated nylon membranes (**b**) during oily wastewater treatment. Conditions: pH = ~6.0, $C_{O\&G0}$ = 100 ± 5 mg/L, $C_{H2O20}$ = 20 mg/L (in the oxidation tests), $V_{emulsion}$ = 1.5 L, T = 25 °C, P = 1 bar

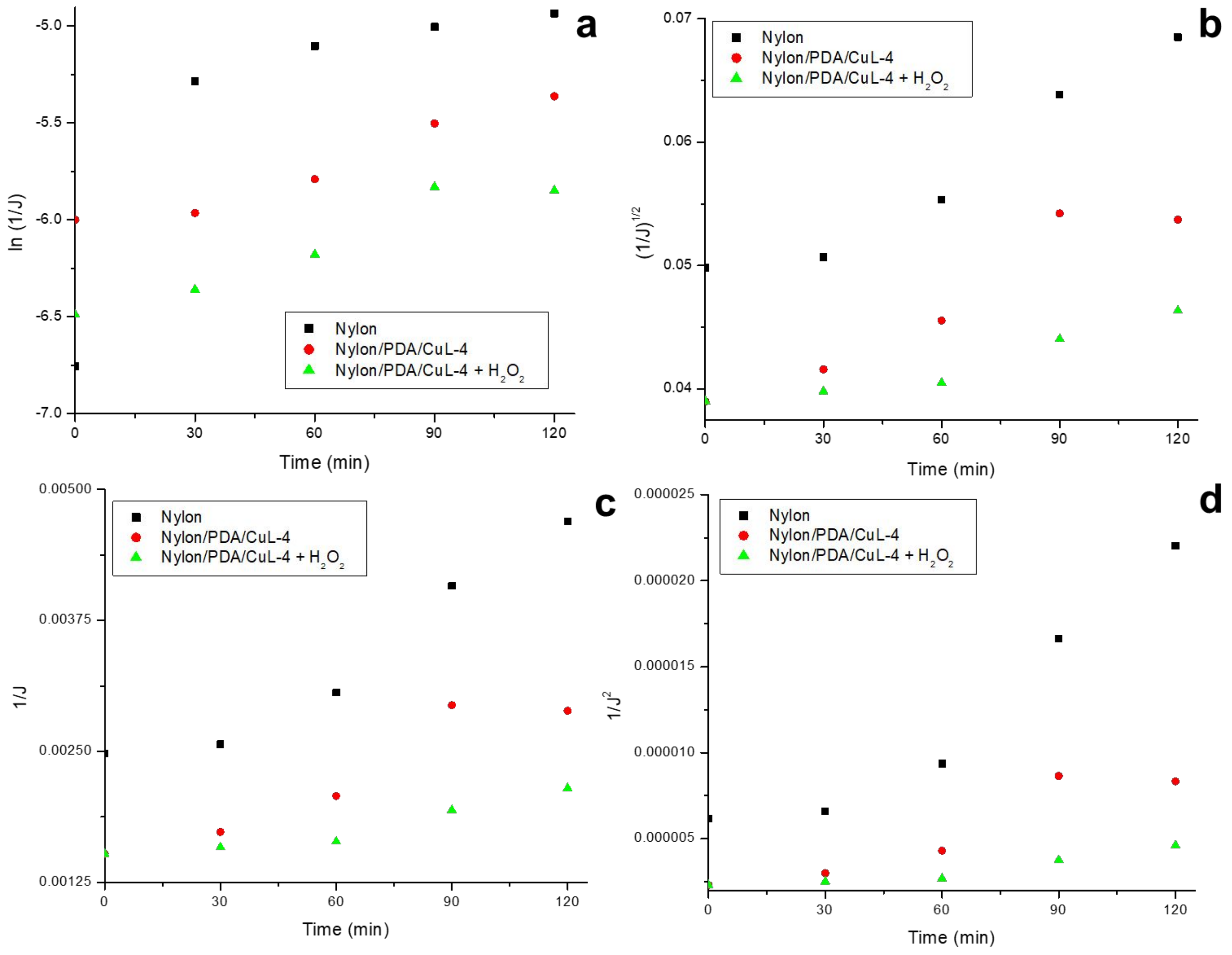


**Figure S16.** Graphs associated with the Hermia models applied to the treatment data with pristine and CuL decorated nylon membranes: BTP (**a**), OP (**b**), BPP (**c**), and e TF (**d**)

**Table S1.** Equations corresponding to Hermia's models.

| model | Equation |
|---|---|
| **BTP** | $ln\left(\frac{1}{J}\right) = ln\left(\frac{1}{J_0}\right) + K\,t$ |
| **OP** | $\left(\frac{1}{J}\right)^{\frac{1}{2}} = (\frac{1}{J_0})^{\frac{1}{2}} + K\,t$ |
| **BPP** | $\left(\frac{1}{J}\right) = \left(\frac{1}{J_0}\right) + K\,t$ |
| **TF** | $\left(\frac{1}{J^2}\right) = \left(\frac{1}{{J_0}^2}\right) + K\,t$ |

**Table S2.** Fouling parameters and $R^2$ for the Hermia fouling models of the pristine and CuL decorated nylon membranes.

| model | parameters | nylon | nylon/PDA/CuL-4 | nylon/PDA/CuL-4 + $H_2O_2$ |
|---|---|---|---|---|
| **BTP** | **K ($h^{-1}$)** | 5.79 x $10^{-3}$ | 6.04 x $10^{-3}$ | 2.98 x $10^{-3}$ |
| | **$R^2$** | 0.9482 | 0.9298 | 0.9223 |
| **OP** | **K ($m/(L^{0,5}\ h^{0,5})$)** | 1.69 x $10^{-4}$ | 1.40 x $10^{-4}$ | 0.632 x $10^{-4}$ |
| | **$R^2$** | 0.9406 | 0.9195 | 0.9155 |
| **BPP** | **K ($m^2/L$)** | 1.98 x $10^{-5}$ | 1.31 x $10^{-5}$ | 0.537 x $10^{-5}$ |
| | **$R^2$** | 0.9313 | 0.9072 | 0.9082 |
| **TF** | **K ($m^4\ h/L$)** | 1.39 x $10^{-7}$ | 0.590 x $10^{-7}$ | 0.195 x $10^{-7}$ |
| | **$R^2$** | 0.9082 | 0.8786 | 0.8927 |
